# Design of Photocatalytic Composite Coatings for Formaldehyde Removal Based on the Visual Effects of Wood Furniture

**DOI:** 10.1002/advs.76906

**Published:** 2026-07-29

**Authors:** Xinyue Wei, Shuyuan Chen, Xinlong Zhou, Han Wang, Wei Guo, Xiaoting Niu, Wei Li, Zhen Ding, Haiyuan Chi

**Affiliations:** ^1^ College of Materials Science and Engineering, Key Laboratory of Bio‐based Material Science and Technology of Ministry of Education Northeast Forestry University Harbin China; ^2^ College of Home and Art Design Northeast Forestry University Harbin China; ^3^ Hebei Key Laboratory of Close‐to‐Nature Restoration Technology of Wetlands, School of Eco‐Environment Hebei University Baoding P. R. China; ^4^ College of Mechanical Engineering Heilongjiang University of Science and Technology Harbin China; ^5^ College of Computer and Control Engineering Northeast Forestry University Harbin China

**Keywords:** coatings, formaldehyde, photocatalytic, visual effects, wood furniture

## Abstract

Constructing photocatalytic composite coatings to remove indoor formaldehyde (HCHO) is an important approach to ensuring a healthy indoor environment. However, most current research focuses primarily on the design of photocatalysts and coatings, while overlooking the impact of applying these coatings to wood surfaces on the visual appearance of furniture. Here, a waterborne polyurethane (WPU) photocatalytic composite coating was developed using four fundamental photocatalyst color systems and applied to four types of wood to examine its influence on color difference and visual appearance. The catalyst's ability and mechanism for efficiently degrading indoor HCHO were verified through material characterization and HCHO degradation experiments. By adjusting the ratios of photocatalysts to construct coatings, both HCHO degradation performance and essential mechanical properties were shown to meet basic requirements. Color‐difference analysis and eye‐tracking data were used to assess how different photocatalytic composite coatings affect the visual perception of various wood types. Intelligent design processes were developed using an XGBoost classifier based on gradient‑boosted decision trees and a Bayesian‑optimized inverse design framework. This system proposes an intelligent design framework for photocatalytic composite coatings for wooden furniture, aiming to provide more methodological references.

## Graphical abstract

This work develops photocatalytic coatings for indoor formaldehyde removal on wood surfaces. It explores coating‐induced color variations and visual perception via eye‐tracking tests. Combined with XGBoost and Bayesian optimization, an intelligent design framework is established to guide the optimization of visually friendly wood photocatalytic coatings.

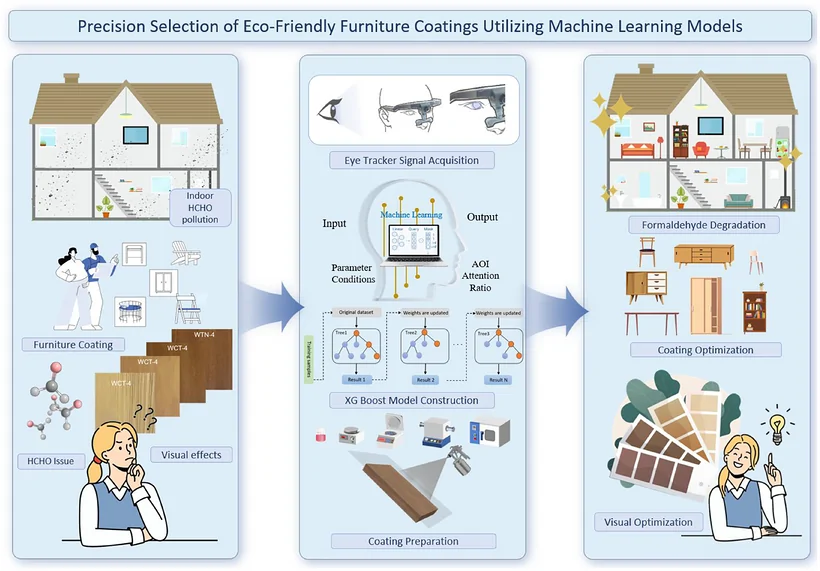

## 1 Introduction

Wood is an intimate “companion” that is inseparable from people's lives and work, and newly acquired furniture brings beauty and convenience to people. At present, artificial boards such as plywood, density board, and particleboard are widely used in furniture materials, and their main adhesives include phenolic resin and urea formaldehyde (HCHO) resin [1]. These resins not only release HCHO generated by reactions during use, but also release unreacted HCHO, becoming the main source of HCHO pollution in indoor air. HCHO is colorless and has a strong irritant odor. It is absorbed by the human body through respiration, skin, and conjunctiva, and quickly dissolves in body fluids and is oxidized to formic acid, causing cascading damage to multiple systems. The hazards of HCHO have surpassed the traditional category of “odor” and become a systemic public health issue involving cell toxicity, immune imbalance, carcinogenic mutations, and reproductive and developmental toxicity [2, 3]. With the improvement of living standards and the increasing public demand for indoor environmental health and working environments, effectively curbing and eliminating the harmful effects of HCHO emissions from wood furniture on human health has become a widely concerned issue [4].

To effectively remove indoor HCHO, researchers have developed and applied a variety of technological approaches, including photocatalytic oxidation, biological and plant‐based adsorption–filtration, activated carbon adsorption, and plasma oxidation [5, 6]. Among these methods, photocatalytic oxidation stands out due to its notable advantages. In this process, light irradiation excites semiconductor catalysts to generate electron–hole pairs, which then form highly oxidative active species on the catalyst surface, enabling efficient degradation of HCHO [7]. Photocatalytic oxidation offers several merits, such as simple operation, catalyst stability, no additional energy consumption, complete pollutant mineralization, and an absence of secondary pollution, making it a major focus in current indoor HCHO‐removal research [8]. However, most photocatalysts are available in powder form with high specific surface areas, which limits their direct application onto wood surfaces for HCHO degradation. To address this challenge, researchers have immobilized powdered photocatalysts onto various substrates to develop photocatalytic coatings for wood furniture [9]. This strategy not only ensures sufficient exposure of the photocatalyst to light, enhancing its catalytic efficiency and activity, but also mitigates issues such as uneven distribution and agglomeration, thereby enabling effective removal of HCHO emitted from wood‐based materials [10, 11]. In addition to improving indoor air quality by degrading HCHO, photocatalytic coatings can also enhance the weather resistance, abrasion resistance, and overall durability of wood furniture, contributing to improved design and functional performance [12]. Therefore, advancing photocatalytic coating technologies is essential for both indoor environmental protection and the optimized design of wood furniture.

Common photocatalytic coatings are typically composed of photocatalysts and carrier materials. The carrier material serves to support and immobilize the photocatalyst, ensuring firm adhesion to the substrate surface. Commonly used carriers include nano‑SiO_2_, waterborne polyurethane (WPU), and epoxy resin [13, 14]. Among them, WPU is a novel polyurethane system that employs water rather than organic solvents as the dispersion medium. By dispersing polyurethane in water to form a uniform emulsion, WPU offers advantages such as nonflammability, non‑toxicity, environmental friendliness, excellent mechanical properties, good compatibility, and ease of modification, making it widely applied in photocatalytic coating preparation. In previous studies, TiO_2_ powder has been incorporated into WPU as a filler to prepare photocatalytic coatings that play a significant role in indoor HCHO degradation [15]. Building on this work, other researchers have further introduced MOFs materials together with TiO_2_ to form composite fillers, thereby enhancing WPU‑based coatings [16]. These improvements not only strengthen the physical properties of the coatings but also increase their photocatalytic degradation performance by improving catalyst adsorption capacity. However, WPU is typically colorless and transparent, and it often exhibits complex coloration when loaded with photocatalysts. When such modified photocatalytic coatings are applied to wood furniture surfaces, they may adversely affect the wood's natural texture and visual quality. To date, research on photocatalytic coatings has primarily focused on coating preparation and catalytic degradation performance, while the impact of these coatings on the texture and visual experience of wood furniture has been largely overlooked.

In this study, we prepared four types of photocatalytic coatings with different colors and applied them to the photocatalytic degradation system of HCHO in different materials of wood. First, we synthesized and characterized four types of photocatalysts, and systematically studied their performance and mechanism of photocatalytic degradation of HCHO. Subsequently, a photocatalytic composite coating was prepared using waterborne polyurethane as the substrate, and its HCHO degradation effect and basic mechanical properties were evaluated. Finally, the impact of photocatalytic composite coatings on wood color values and visual perception was analyzed using machine learning methods. Based on the above research, the correlation mechanism between the degradation performance of the photocatalytic composite coating, the color difference of the coating, and the visual experience was established. This provides a theoretical basis for the reverse optimization design of photocatalytic coatings and the aesthetic research of wooden furniture.

## 2 Results and Discussion

### 2.1 Study of the Photocatalytic Degradation of HCHO by Catalysts

According to the reference, four modified Ti‐based MOFs catalysts with different colors were prepared by a hydrothermal method: white TiO_2_@MIL‐125, light‐yellow TiO_2_@NM‐120, light‐green Cu_0.20_Ti‐NM, and black Cu‐TiO_2_/C. TiO_2_@MIL‐125 exhibits a disc‐shaped morphology with TiO_2_ uniformly dispersed on the MIL‐125 surface (Figure 1a). The sample surface is smooth, elemental mapping shows Ti, O, N, and C, with TiO_2_ particles visible as surface aggregates (Figure 1b and Figure S2). XRD patterns display characteristic peaks at 2θ ≈ 6.8°, 9.7°, 11.7°, 16.6°, 17.9°, and 19.5°, corresponding to the reflections of MIL‐125 and TiO_2_, confirming the coexistence of both crystal phases (Figure 1i) [17]. TiO_2_@NM‐120 is structurally similar to TiO_2_@MIL‐125, exhibiting a disc‐shaped morphology with TiO_2_ uniformly distributed across the surface (Figure 1c). Elemental mapping shows Ti, N, O, and C in aggregated states consistent with surface TiO_2_ (Figure 1d). Unlike TiO_2_@MIL‐125, the surface of TiO_2_@NM‐120 is relatively rough, attributable to defect structures. The formation of the defect structure has been confirmed through correlation analysis [16, 18]. XRD patterns show characteristic peaks at 2θ ≈ 6.8°, 9.7°, 11.7°, 16.6°, 17.9°, 25.34°, 38.22°, 48.04°, and 54.92°, indicating the coexistence of TiO_2_ and NM‐120 crystalline phases (Figure 1i) [19]. Unlike the previous two catalysts, Cu_0.20_Ti‐NM exhibits an isolated disc‐shaped morphology (Figure 1e), with a uniform distribution of Cu, Ti, O, C, and N (Figure 1f). The characteristic peaks observed at 2θ ≈ 6.8°, 9.7°, 11.7°, 16.6°, and 17.9° correspond to NM‐specific reflections (Figure 1i), indicating that the Cu has not formed separate crystalline phases within the catalyst. After pyrolysis, the Cu‐TiO_2_/C catalyst derived from Cu‐based Ti‐MOFs retains its disc‐shaped morphology with slight shrinkage in size (Figure 1g). Elemental mapping shows C, N, O, Ti, and Cu uniformly distributed throughout the structure (Figure 1h). XRD displays characteristic peaks at 2θ ≈ 25.2°, 43.3°, and 50.4°, consistent with the presence of crystalline TiO_2_, amorphous carbon, and Cu_2_O phases (Figure 1i) [20]. Based on the SEM images and XRD patterns of white TiO_2_@MIL‐125, light‐yellow TiO_2_@NM‐120, light‐green Cu_0.20_Ti‐NM, and black Cu‐TiO_2_/C, comparison with the literature confirms the successful synthesis of four distinct, differently colored catalysts.

**FIGURE 1:**
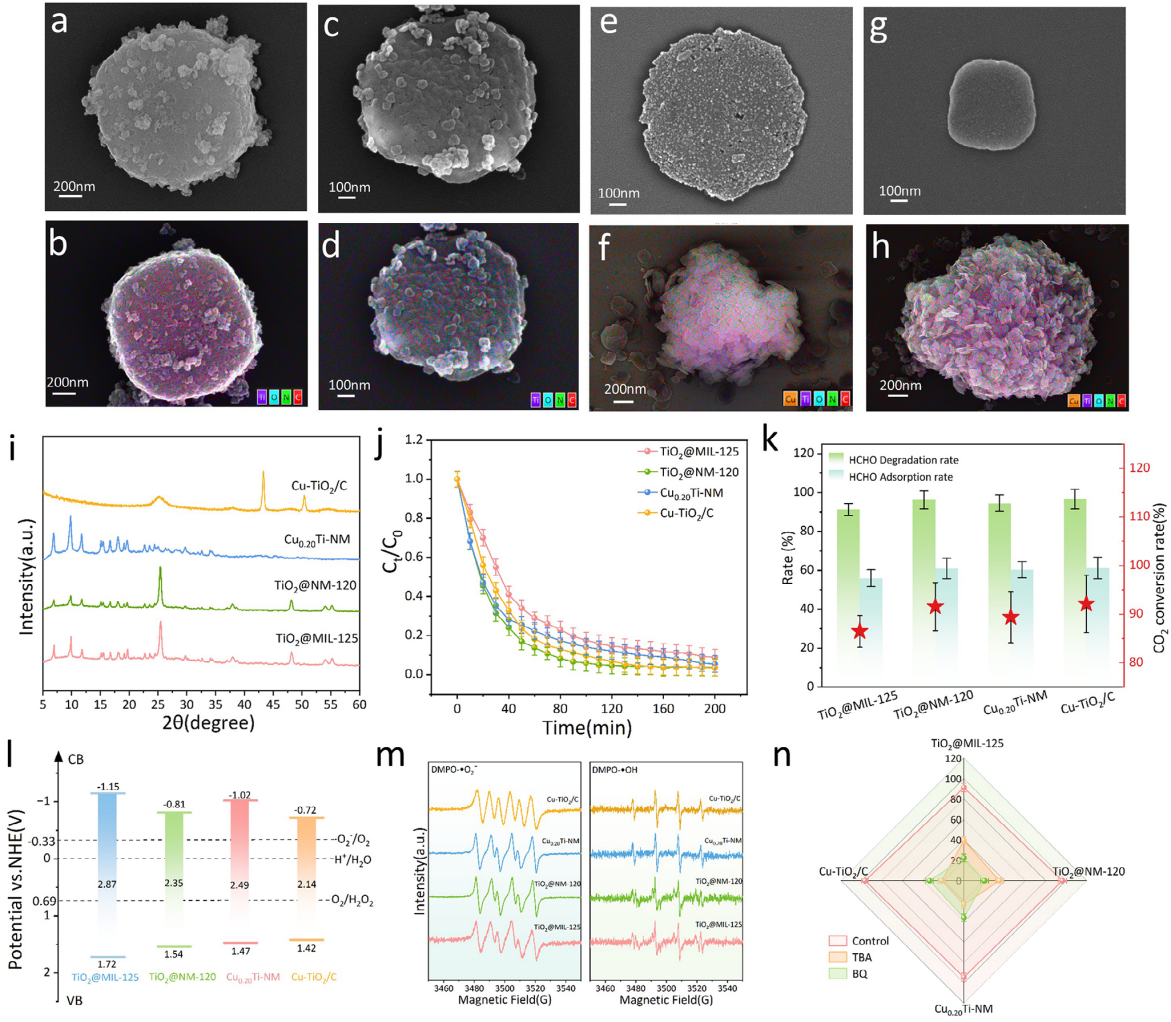
The SEM images and EDS element mappings of (a, b) TiO_2_@MIL‐125; (c, d) TiO_2_@NM‐120; (e, f) Cu_0.20_Ti‐NM; (g, h) Cu‐TiO_2_/C; (i) The XRD patterns; (j) The photocatalytic degradation rate curves; (k) The photocatalytic degradation rate and CO_2_ conversion rate during the photocatalytic reactions; (l) The band structures of samples. (m) The EPR spectra for DMPO •O_2_^−^ and DMPO •OH under light irradiation; (n)Free radical scavenging experiment under illumination.

Measure the photocatalytic degradation efficiency of the four catalysts for HCHO under sealed conditions to evaluate their photocatalytic activity (Figure 1j). When any of the four catalysts was added, the HCHO concentration dropped rapidly and reached a relatively stable level after about 2 h of irradiation. Under light exposure, TiO_2_@MIL‐125, TiO_2_@NM‐120, Cu_0.20_Ti‐NM and Cu‐TiO_2_/C achieved degradation efficiencies of approximately 91.25%, 96.29%, 94.57% and 96.58%, respectively. A HCHO removal rate exceeding 90% shows that all four selected catalysts enable rapid degradation of HCHO, supporting their suitability for incorporation into coating materials for photocatalytic HCHO removal. To eliminate interference from catalyst adsorption, the adsorption performance of the four catalysts was measured under identical conditions in the dark. Figure 1k *left* shows the HCHO removal rates for TiO_2_@MIL‐125, TiO_2_@NM‐120, Cu_0.20_Ti‐NM and Cu‐TiO_2_/C are 56.12%, 61.55%, 60.34% and 61.24%, respectively. The adsorption‐based removal of HCHO is far lower than the catalytic degradation observed under irradiation, indicating that the four catalysts primarily rely on photocatalytic oxidation to degrade HCHO. The mineralization efficiencies measured for the four photocatalytic systems further confirm that HCHO is oxidatively degraded (Figure 1k *right*). The conversion rates of HCHO to CO_2_ are TiO_2_@MIL‐125 (86.45%), TiO_2_@NM‐120 (91.55%), Cu_0.20_Ti‐NM (89.36%), and Cu‐TiO_2_/C (92.11%), indicating the production of small‐molecule CO_2_ during oxidation. Together, these results show that all four catalysts effectively activate photocatalysis and fully oxidize HCHO to CO_2_.

To further demonstrate the photocatalytic performance of the four catalysts, we conducted a series of experiments. UV–vis diffuse reflectance spectra indicate that all four catalysts exhibit some visible‐light absorption (Figure S3), which is important for their photocatalytic activity [21]. From the Tauc plot in Figure S4, the bandgaps of TiO_2_@MIL‐125, TiO_2_@NM‐120, Cu_0.20_Ti‐NM, and Cu‐TiO_2_/C are 2.87, 2.35, 2.49, and 2.14 eV, respectively. X‐ray photoelectron spectroscopy (XPS) valence band measurements (Figure S5) place the valence‐band positions of TiO_2_@MIL‐125, TiO_2_@NM‐120, Cu_0.20_Ti‐NM, and Cu‐TiO_2_/C at 1.72, 1.54, 1.47, and 1.42 eV, respectively. Using the bandgap and valence band data, we calculated the band structure diagrams for the four catalysts (Figure 1l). Calculations show that the conduction bands of all four catalysts lie below the O_2_/^•^O_2_^−^ reduction potential (−0.33 eV), making formation of ^•^O_2_^−^ thermodynamically favorable and enabling photocatalytic oxidation of HCHO [22]. As primary reactive species, the generated ^•^O_2_^−^ can undergo subsequent chain reactions to produce ^•^OH, and the combined action of ^•^O_2_^−^ and ^•^OH drives the synergistic degradation of HCHO. The relevant studies were verified through DFT calculations [5]. EPR spectra confirm that superoxide radicals convert into hydroxyl radicals via chain reactions (Figure 1m). As shown, signals for both ^•^O_2_^−^ and ^•^OH appear in the photocatalytic systems of all four catalysts, demonstrating their presence as active species for HCHO degradation [23]. Quenching experiments further support their synergistic role: selectively scavenging either ^•^OH or ^•^O_2_^−^ markedly reduces HCHO degradation efficiency for all four catalysts (Figure 1n and Figure S6). These results indicate that photocatalytic degradation of HCHO by the catalysts proceeds through the combined action of ^•^O_2_^−^ and ^•^OH. Based on the above experiments, we have successfully synthesized modified Ti‐MOF photocatalysts in four distinct colors (white TiO_2_@MIL‐125, light‐yellow TiO_2_@NM‐120, light‐green Cu_0.20_Ti‐NM, and black Cu‐TiO_2_/C) and demonstrated that under visible light they generate both ^•^O_2_^−^ and ^•^OH. These reactive species drive the photocatalytic oxidation of HCHO into smaller molecules.

### 2.2 Study on the Performance of Photocatalyst‐Based Functional Coatings

We evaluated the applicability of the four photocatalysts for indoor HCHO control by incorporating them into wood coatings. Each catalyst was dispersed into a waterborne polyurethane (WPU) formulation to produce environmentally friendly, photocatalytic composite coatings, namely WPU‐TiO_2_@MIL‐125 (WTM‐X), WPU‐TiO_2_@NM‐120 (WTN‐X), WPU‐Cu_0.20_Ti‐NM (WCT‐X), and WPU‐Cu‐TiO_2_/C (WCC‐X). The composite coatings were successfully sprayed onto wooden substrates, and their photocatalytic performance for degrading indoor HCHO was then assessed (Figure 2a). WPU alone cannot effectively remove HCHO, so the coating itself does not contribute significantly to HCHO reduction. Adding a photocatalyst to WPU produces a marked improvement in HCHO removal. Prior to photocatalytic testing, we exposed four photocatalytic composite coatings to dark conditions and observed a slight decrease in HCHO concentration. The decline correlated with the amount of photocatalyst added, indicating that while the photocatalyst adsorbs some HCHO, adsorption is not the primary mechanism for oxidation and degradation. Under illumination, the HCHO removal rate increased rapidly. As photocatalyst loading rose, removal efficiency first improved and then declined — a pattern observed for all four composite coatings (Figure 2b and Figure S7). This demonstrates that photocatalyst content is critical for removal performance. The optimal HCHO removal occurred at a photocatalyst loading of 4%, likely due to effective dispersion of the photocatalyst within the WPU matrix. The CO_2_ production rate follows the same trend as HCHO degradation, which indicates two key points. First, the photocatalytic composite coatings effectively oxidize and degrade HCHO into small‐molecule CO_2_, confirming the photocatalyst's role in HCHO decomposition [24]. Second, the photocatalyst itself is the primary agent responsible for HCHO degradation in these coatings, and its loading level determines the effective activity and efficiency of the degradation process. This demonstrates the photocatalytic composite coating's effective degradation of HCHO. At photocatalyst loadings of 3–5%, the coating can achieve over 90% HCHO removal within 6 h.

**FIGURE 2:**
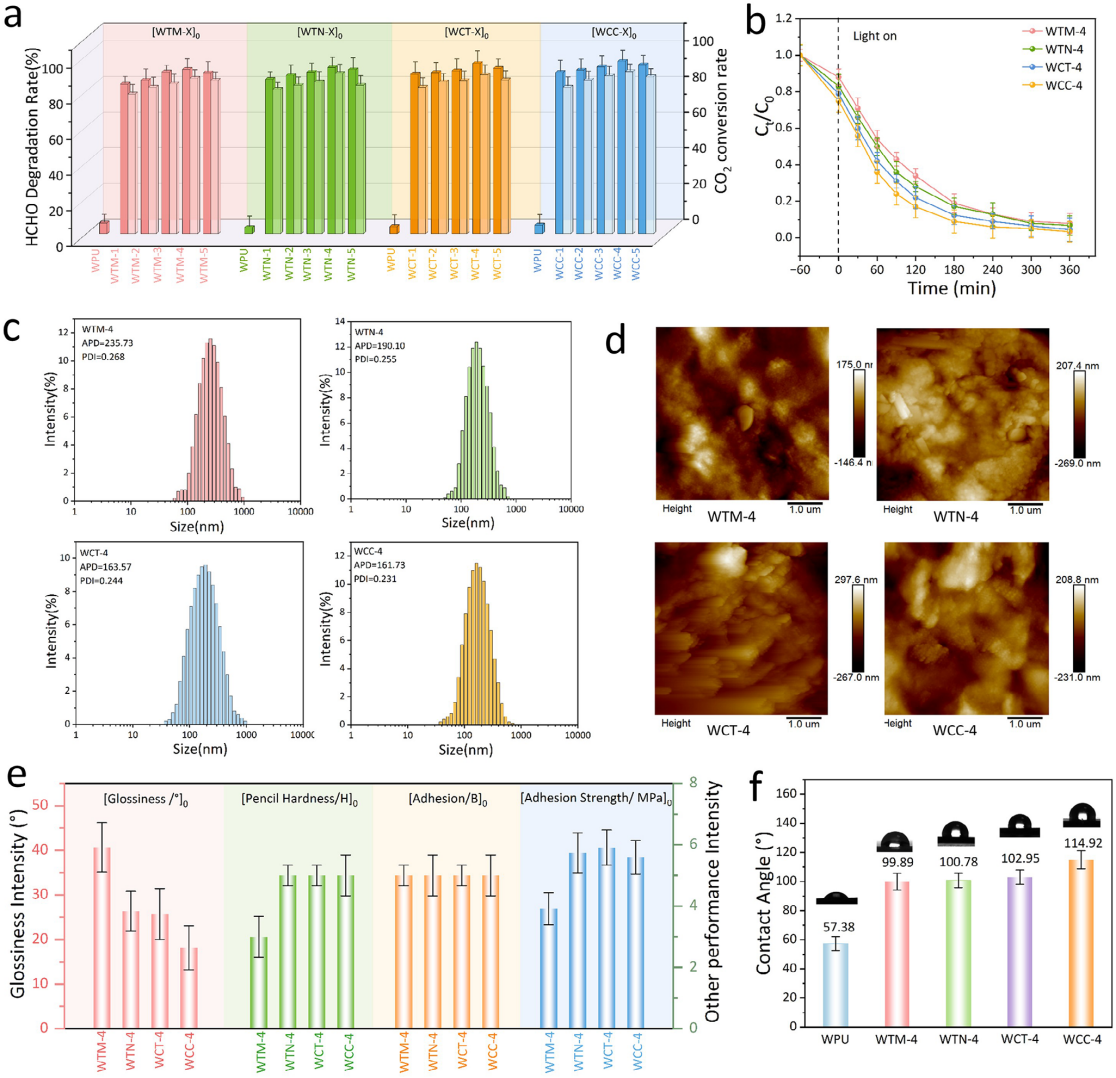
(a) Photocatalytic degradation activity of coating; (b) Photocatalytic degradation rate; (c) Particle sizes and size distributions of sample dispersions; (d) AFM images of coating; (e) Glossiness, pencil hardness, adhesion and adhesion strength of coatings; (f) Water contact angle of the coating.

Dynamic light scattering (DLS) analysis was used to evaluate the photocatalyst's effect on the stability of the WPU dispersion (Figure 2c and Figures S8 and S9). Adding the photocatalyst increases the average particle size of the composite, attributable to stable interfacial interactions between the WPU and photocatalyst nanoparticles [25]. Consistent with the trend in HCHO degradation efficiency, the average particle size of the photocatalytic composite coating dispersion increases with photocatalyst loading and peaks at 4%. Beyond 4% loading, the average particle size decreases, likely due to photocatalyst aggregation from excessive addition. The same pattern observed across four different composite coatings indicates this is an intrinsic behavior rather than a property of a single photocatalyst. This suggests the photocatalytic degradation of HCHO is closely related to the dispersion's average particle size: when particle size is at its maximum, the number of effective photocatalytic degradation sites is maximized, which enhances HCHO degradation [26]. The average particle size of the photocatalytic composite coating dispersion correlates positively with the coating's surface roughness, as particle size increases, roughness increases as well. Therefore, atomic force microscopy (AFM) was used to characterize the surface roughness of the coatings at the optimal formulation. The AFM images show that adding the photocatalyst increases the number of irregular protrusions on the WPU surface, consistent with the observed increase in average particle size (Figure 2d and Figure S10). The Rq values, which quantify surface roughness, for the four photocatalytic composite coatings are 95.3 nm (WTM‐4), 65.2 nm (WTN‐4), 63.4 nm (WCT‐4), and 60.9 nm (WCC‐4), respectively. Compared with the WPU coating (Rq = 6.86 nm), photocatalytic composite coatings exhibit substantially increased surface roughness. This enhanced roughness promotes diffuse light scattering, thereby improving the practical matte appearance of the coatings [27, 28]. Moreover, even at the highest measured roughness, the photocatalytic composite coatings remain within the acceptable roughness range for real wood applications, indicating that formulations with different photocatalyst loadings have good practical potential.

To evaluate the practicality of the photocatalytic composite coatings, their basic properties—gloss, pencil hardness, adhesion, and adhesion strength—were examined. Figure S11 shows the gloss values for coatings with different photocatalyst loadings. As expected, increasing the photocatalyst content reduces gloss, attributable to diffuse light scattering from the catalyst's inherently rough surface [29]. All prepared photocatalytic composite coatings exhibit gloss values between 15 and 60, corresponding to a matte finish. This range meets the gloss requirements for wood furniture surfaces and supports their suitability as matte coatings. Pencil hardness testing was employed to evaluate the durability and long‐term practicality of the photocatalytic composite coatings (Figure S12). Results show that hardness increased from 1H to 5H as photocatalyst loading increased. This improvement is partly attributable to the higher intrinsic hardness of the photocatalyst relative to WPU and partly to strong covalent and hydrogen‐bonding interactions formed between the photocatalyst and WPU during synthesis, which significantly enhance the coatings’ mechanical properties [30]. Adhesion was assessed by the cross‐cut (paint film scratch) test; results are shown in Figure S13. The WPU coating alone scored 2B, while adding photocatalysts improved adhesion, reaching as high as 5B for the best formulations. Interfacial strength between wood and the coatings was quantified by peel testing. Pure WPU exhibited an adhesion strength of 1.51 MPa, and incorporation of various photocatalysts increased this value, with the highest formulations achieving 5.98 MPa level (Figure S14). This improvement is attributed to the photocatalytic additives’ porous structure and high specific surface area, which promote physical interlocking with waterborne polyurethane chains and strengthen the interface [31]. However, excessive photocatalyst loading can reduce adhesion, likely due to nanoparticle aggregation or precipitation at high concentrations. We compared the mechanical properties of several types of photocatalytic composite coatings (Figure 2e). Although photocatalytic performance varied among formulations, all samples met the basic requirements for wood coatings in terms of gloss, pencil hardness, adhesion, and bonding strength, demonstrating the practical potential of surface‐applied photocatalytic composite coatings. Additionally, contact angle measurements of the four coating types indicate that the addition of photocatalysts increases the contact angle, demonstrating enhanced hydrophobicity. This is a key factor in maintaining coating stability in humid environments (Figure 2f) [32]. The experiments demonstrate that all four colored photocatalysts can be used to produce photocatalytic composite coatings. At photocatalyst loadings of 3%–5%, the coatings achieve suitable HCHO degradation, surface roughness, gloss, and hardness for use as wood furniture coatings. These results provide a data foundation for subsequent furniture design.

### 2.3 Intrinsic Relationship Typical Wood‐Photocatalytic Composite Coating‐Visual Effects

To clarify the influence of photocatalytic composite coatings of different colors on the visual effects of wood furniture, we conducted a series of quantitative tests and sensory evaluations. Based on the basic performance data of four coating colors (each containing five levels of photocatalyst content), we selected Red Oak, European Ash, Cherrywood, and Sapelli as four representative furniture materials for the visual‑effect experiments. In the quantitative visual‑effect experiment, we objectively analyzed the color of wood treated with photocatalytic composite coatings by measuring their L*, a* and b* values, generating quantitative data on how these coatings influence wood coloration. L* represents changes in lightness, a* represents shifts along the red‐green axis, and b* represents shifts along the yellow‐blue axis [33]. The L*, a*, and b* values of the four wood types treated with different photocatalytic composite coatings are shown in Figure 3. As is well known, traditional WPU coatings are colorless and transparent, so coatings without photocatalysts can essentially reflect the initial color values of the woods. The consistent initial L*, a* and b* values across the different photocatalytic composite coatings applied to the four wood types further confirm the reliability of the data. The differences in the initial L*, a* and b* values among the four wood types also support the use of L*, a* and b* values as a reasonable method for analyzing color differences in wood. The effects of the white WTM‐X photocatalytic composite coating on the L*, a* and b* values of different wood types are shown in Figure 3a. The results indicate that white WTM‐X generally increases the L* value of wood while reducing the a* and b* values. An increase in L* value makes the wood surface appear brighter, whereas decreases in a* and b* values result in a purer, less saturated color. This high‑brightness, low‑saturation appearance typically conveys a fresh, clean, and modern visual impression. The pale yellow WTN‐X generally the L* and b* values of the wood while reducing a* value (Figure 3b). A higher L* value makes the wood surface appear brighter, a moderate increase in b* value (greater yellowness) adds a* warm and natural tone, and a decrease in a value (reduced redness) prevents the color from becoming overly red or purplish, resulting in a more balanced overall hue. This combination of high brightness, warm tones, and low saturation helps create a cozy, comfortable atmosphere while preserving the natural aesthetic of the wood [34, 35]. Similar to the effect of WTN‐X on wood color values, the light green WCT‐X also increases the L* and b* values while reducing a* value, producing comparable overall effects (Figure 3c). The black WCC‐X simultaneously reduces the L*, a*, and b* values of the wood (Figure 3d). Lower L* values (reduced brightness) give the surface a more stable and subdued appearance, while decreases in both b* (yellowness) and a* (redness) shift the color toward neutral gray or cooler tones. This combination of low brightness, low saturation, and cool hues helps create a refined, modern, and minimalist visual style. In addition, as the catalyst content increases, the L*, a*, and b* values exhibit a linear increasing or decreasing trend, indicating that the influence of the catalyst on color values remains stable. In addition, as the catalyst content increases, the L*, a*, and b* values exhibit clear linear trends, indicating that the influence of the photocatalytic composite coatings on wood color remains stable within the 0%–5% catalyst content range.

**FIGURE 3:**
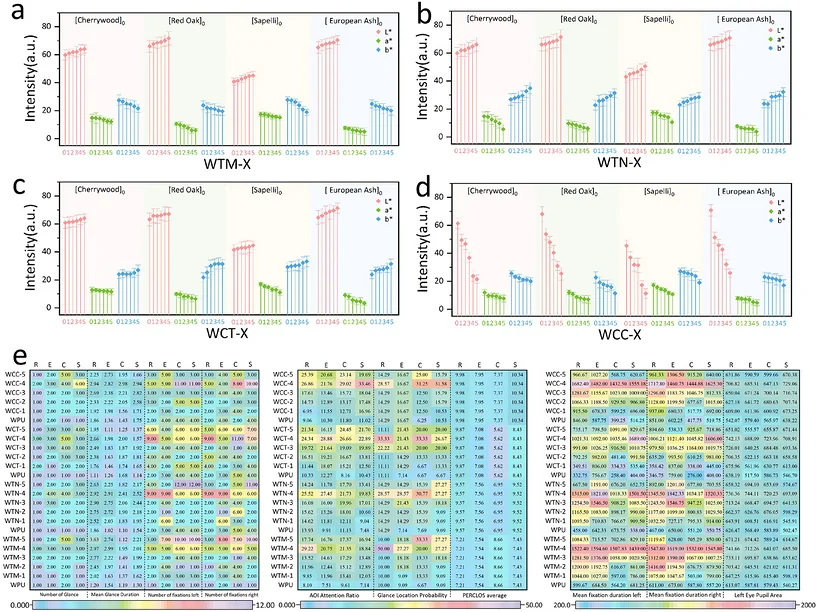
(a) Lab value of the WTM‐X coating; (b) Lab value of the WTN‐X coating; (c) Lab value of the WCT‐X coating; (d) Lab value of the WCC‐X coating; (e) Eye Tracker Parameter Heatmap.

In the sensory evaluation of visual effects, we assessed the appearance of different woods coated with photocatalytic composite coatings using participants’ eye‐tracking data. Twenty participants took part in the experiment, and their data were comprehensively analyzed. To examine how different colors influence visual responses, we divided the eye‐tracking images of wood samples with varying photocatalyst contents into six areas of interest (AOIs) and calculated the corresponding interest‐value parameters for each AOI. The results are shown in Figure 3e and Tables S1–S8. Visually, the overall colors of WTM‐X and WCT‐X appear lighter, with WTM‐X trending brighter and WCT‐X appearing softer. This effect becomes more pronounced as catalyst content increases. Hotspot map results indicate that both brighter and softer visual effects correspond to higher hotspot values, producing the most comfortable gaze response at WTM‐4 and WCT‐4. Beyond this point, the reduced gaze effect further supports this conclusion (Figures S15 and S16). In contrast, WTN‐X and WCC‐X gradually darken, achieving stronger fixation effects by enhancing the wood's inherent deep structure. However, their fixation values also decline after surpassing an optimal ratio, indicating the presence of an optimal observation range (Figures S17 and S18). This pattern appears consistently across all four wood types, suggesting that the phenomenon is systematic rather than incidental. Eye‐tracking data on pupil dilation further support these findings: greater dilation, corresponding to longer fixation times, reflects stronger visual stimulation caused by color changes [36]. To further clarify how different colors influence visual effects, we compared eye‐tracking patterns for coatings of various colors across the four wood types. As shown in Figure S19 and Tables S9–S12, although the woods differ visually, the hotspot trajectories and visual‐attention patterns generated by the coatings remain consistent. In all cases, the relationship follows WCC‐4> WCT‐4> WPU > WTM‐4> WTN‐4. This indicates that, within the same wood type, WCC‐4 gains the longest gaze duration due to its deeper visual effect. It enhances wood color saturation and strengthens its three‐dimensional appearance, making it suitable for home display areas. WCT‐4 shows the second‐highest fixation time because it falls within a spectrally sensitive range, making it appropriate for home office or study areas. Although WTM‐4 receives less attention, its relatively high brightness can still create a comfortable living atmosphere, making it suitable for leisure and entertainment spaces. In contrast, WTN‐4 receives the least attention among the four, suggesting that it is the least visually stimulating under the same conditions and is therefore suitable for home rest areas [37]. Based on the above experimental results, we preliminarily explored the color and visual sensory differences produced by photocatalytic composite coatings on various wood types and proposed initial application scenarios for different catalysts according to these visual responses. However, further work is needed to establish the fundamental properties of photocatalytic composite coatings and to clarify the intrinsic relationship between color and visual effects.

### 2.4 Design of Photocatalytic Composite Coatings Based on Machine Learning Methods

To explore the intrinsic relationship between typical wood‐based photocatalytic composite coatings and their visual effects, we designed the experimental approach shown in Figure 4a. Basic quantitative data‐such as wood type, proportion of photocatalytic composite coating, HCHO degradation performance, fundamental coating properties, coating‐induced changes in wood color difference (L*, a*, and b*), and sensory data obtained from eye‐tracking experiments‐were collected as the initial dataset. Two machine learning models, an XGBoost classifier based on gradient‐boosted decision trees and an inverse‐design framework based on Bayesian optimization, were employed [38]. First, these models were used to establish classification relationships between the quantitative data and sensory responses. Then, the inverse‐design method was applied to determine the optimal catalyst type and formulation ratio under specific color difference (L*, a*, and b*) requirements and HCHO degradation efficiency. This provides guidance for the intelligent design of photocatalytic composite coatings and their application in the wood furniture industry.

**FIGURE 4:**
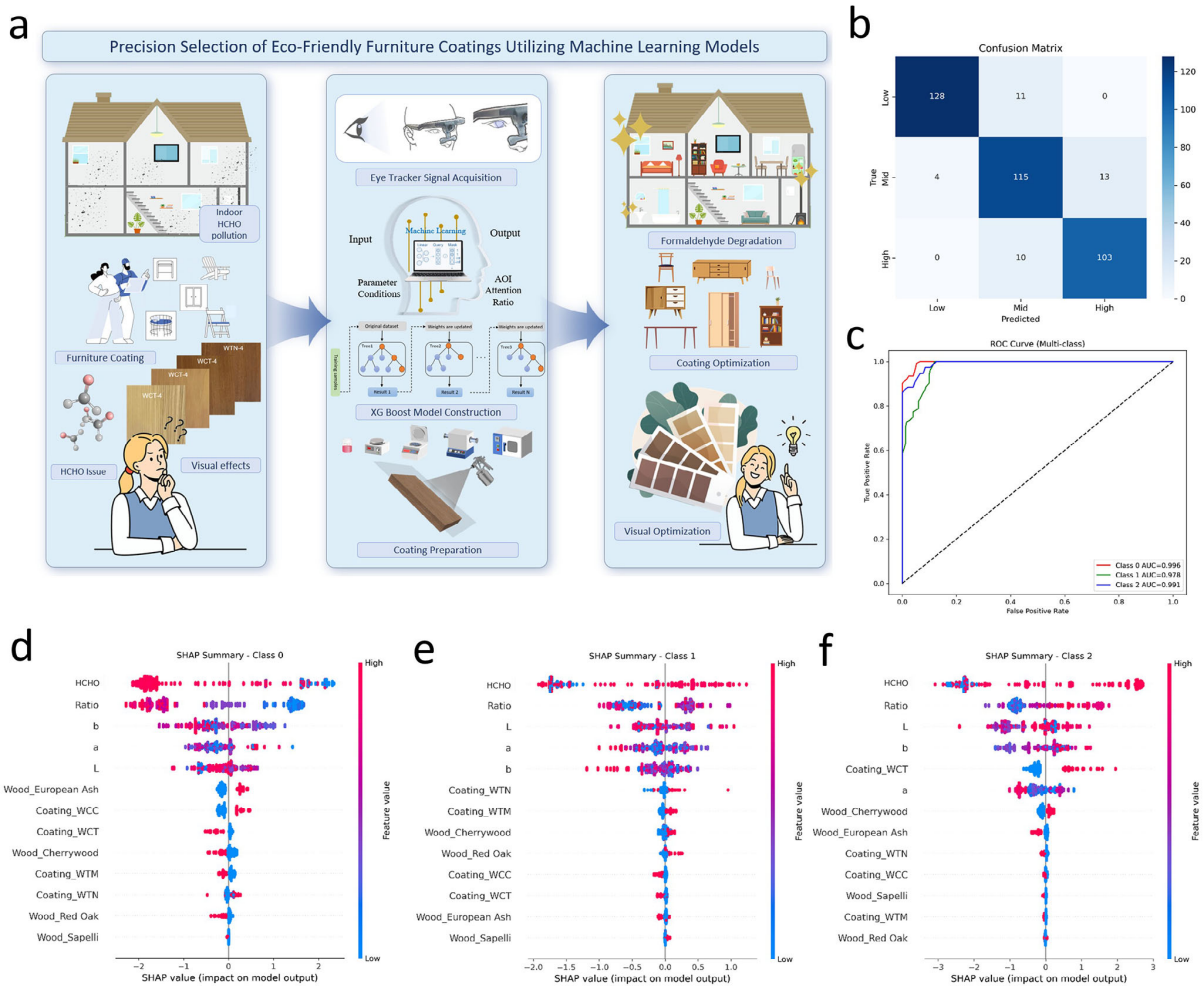
(a) Schematic diagram for optimizing eco‐friendly furniture coatings using machine learning models; (b) Confusion matrix; (c) ROC curve; (d) Low‐level classification of SHAP values; (e) Mid‐level classification of SHAP values; (f) High‐level classification of SHAP values.

In the XGBoost Classifier, we used photocatalytic composite coating data to map the usage characteristics of wood furniture to the attention levels obtained from eye‐tracking experiments. The model inputs included discrete material attributes such as wood substrate type and coating type; quantitative coating‐formulation parameters; L*, a*, and b* chromaticity values of the coated surfaces; and functional indicators such as HCHO degradation efficiency. The attention levels of 20 participants were determined using metrics including average gaze duration and gaze density, and were categorized into low, medium, and high levels. Model performance was evaluated using the confusion matrix and test‐set classification accuracy. The confusion matrix indicates that the model reliably distinguishes among low, medium, and high attention levels (Figure 4b). The method achieved a classification accuracy of 0.901 and excellent recall, precision, and F1‐score, thereby demonstrating excellent performance [39]. Low‐attention samples achieve the highest recognition accuracy (92%), and high‐attention samples are never misclassified as low‐attention, suggesting that highly salient surfaces exhibit clear boundary characteristics in the feature space. ROC analysis further shows that the model attains a true‐positive rate close to 1 under very low false‐positive conditions, indicating that visual saliency features, such as luminance contrast, color saturation, and texture variation, are strongly separable and readily captured by the algorithm (Figure 4c) [40]. To further interpret the model's decision‐making process, SHAP analysis was applied to quantify the contribution of each input feature to the attention‐level classification (Figure 4d–f). The results show that chromaticity components (brightness and color), specific formulation ratios, and degradation efficiency play major roles in prediction [41]. Among the three attention categories, HCHO degradation efficiency is the most influential factor, consistent with our prior analyses. High degradation efficiency corresponds to higher photocatalyst concentrations, which cause stronger alterations to the original wood color, while low degradation efficiency corresponds to lower photocatalyst concentrations and minimal impact on color. Beyond the relationship between degradation efficiency and the color‐difference ratio across different wood types, the contributions of single formulation ratios or individual color‐difference features to attention classification are relatively small. This indicates that more complex, multi‐factor interactions exert a stronger influence on attention‐level prediction. Overall, the results show that for the existing samples, the model achieves high accuracy, enabling effective prediction of human attention levels based on color‐difference values and the photocatalytic HCHO‐degradation efficiency.

Based on the known HCHO‐degradation efficiency and color‐difference parameters, we constructed a reverse‐design approach using Bayesian optimization to infer the selected wood type, catalyst type, and catalyst ratio. To address the non‐uniqueness inherent in the inverse‐design problem, we introduced a generalization‐extension strategy for unseen ratios during the optimization process and adopted a leave‐one‐ratio‐out validation method [42, 43]. The results (Figures S20 and S21) show that Bayesian optimization can identify one or more combinations of photocatalytic composite‐coating parameters and wood parameters within a limited search budget. These combinations produce color‐difference values and HCHO‐degradation efficiencies predicted by the surrogate model that align closely with the target values. Although in some cases the optimal solution does not fully recover the true wood or coating category, its predicted outputs remain highly consistent with the desired outputs. This demonstrates the feasibility and practicality of this reverse engineering approach. Based on formaldehyde degradation efficiency and color difference parameters, we can reverse‐engineer the approximate range of optimal wood species, catalyst types, and ideal formulation ratios. This method allows us to narrow down the optimal solution within a broad range, thereby enabling precise formaldehyde treatment and a more efficient coating process. In summary, we have established a link between “furniture coating colors” and “human visual psychology” through machine learning, and proposed a framework‐based design concept (Figure S22). However, due to the limited size of the dataset, the inverse design framework established using XGBoost and Bayesian optimization is merely an exploratory proof‐of‐concept study and does not yet possess universal applicability or strong generalization capabilities. We will continue to explore this in future research.

## 3 Conclusions

This study successfully synthesized four colors of photocatalytic composite coatings and applied them, through an intelligent‑design framework, to the indoor HCHO‑degradation process of wood furniture. Four structurally similar photocatalysts were selected, and their degradation performance and mechanisms were verified. A photocatalytic composite coating was then formulated with WPU, ensuring effective HCHO removal, and its degradation performance and basic properties were evaluated across different catalyst concentrations. The coatings were subsequently applied to various wood types to examine their effects on wood color difference and visual perception. Finally, machine‑learning models were developed to predict sensory experience based on color‑difference values and HCHO‑degradation efficiency, as well as to reverse‑design the optimal wood type, catalyst, and catalyst ratio from these target outputs. This work provides a framework for the intrinsic relationship between wood‐based photocatalytic composite coatings and visual effects, offering a methodological reference for the targeted design of photocatalytic coatings in wooden furniture applications.

## Author Contributions

**Xinyue Wei**: Conceptualization, Methodology, Data curation, Investigation, Visualization, Formal analysis, Writing – original draft, Writing – review & editing. **Shuyuan Chen**: Investigation. **Xinlong Zhou** and **Han Wang**: Formal analysis. **Wei Guo**: Validation. **Xiaoting Niu**: Resources, Supervision, Writing – review & editing. **Wei Li**: Supervision. **Zhen Ding**: Methodology, Data curation. **Haiyuan Chi**: Writing – original draft, Formal analysis, Methodology, Writing – review & editing. All authors reviewed the manuscript.

## Consent Statement

No written consent has been obtained from the participants as there is no participant identifiable data included in this report.

## Conflicts of Interest

The authors declare no conflicts of interest.

## Supporting information




**Supporting File 1**: advs76906‐sup‐0001‐SuppMat.docx.

## Data Availability

The data that support the findings of this study are available on request from the corresponding author. The data are not publicly available due to privacy or ethical restrictions.
