# Supplementary material for: Design of Photocatalytic Composite Coatings for Formaldehyde Removal Based on the Visual Effects of Wood Furniture
Source: Adv Sci (Weinh). 2026 Jul 29:e76906. Online ahead of print. doi: 10.1002/advs.76906 (PMC13418045; doi:10.1002/advs.76906)
Supplement: Supplementary file 1 — Supporting File 1: advs76906‐sup‐0001‐SuppMat.docx. [file ADVS-9999-e76906-s001.docx]

**Supplementary Information**

Design of Photocatalytic Composite Coatings for Formaldehyde Removal Based on the Visual Effects of Wood Furniture

*Xinyue Wei*^a^*,* *Shuyuan Chen*^a^*, Xinlong Zhou*^b^*,* *Han Wang*^c^*, Wei Guo*^d^*, Xiaoting Niu*^a,b^*^*^, Wei Li*^a^*^*^, Zhen Ding* ^e^*^*^, Haiyuan Chi* ^c^*^*^*

^a^ Key Laboratory of Bio-based Material Science and Technology of Ministry of Education, College of Materials Science and Engineering, Northeast Forestry University, Harbin 150040, China

^b^ College of Home and Art Design, Northeast Forestry University, Harbin 150040, China

^c^ Hebei Key Laboratory of Close-to-Nature Restoration Technology of Wetlands, School of Eco-Environment, Hebei University, Baoding 071002, PR China

^d^ College of Mechanical Engineering, Heilongjiang University of Science and Technology, Harbin 150020, China

^e^ College of computer and control engineering, Northeast Forestry University, Harbin 150040, China

* Corresponding author.

E-mail: [xiaotingniu@nefu.edu.cn](mailto:xiaotingniu@nefu.edu.cn) (X.-T. Niu)

liwei820927@nefu.edu.cn (W. Li)

[dingzhen@nefu.edu.cn](mailto:dingzhen@nefu.edu.cn) (Z. Ding)

Chihaiyuan@hbu.edu.cn ([H.-Y](https://webofscience.clarivate.cn/wos/author/record/1894221). Chi)

**Table of Contents.**

**Fig. S1.** The schematic illustration of the photocatalytic test..

**Fig. S2.** The EDS element mappings.

**Fig. S3.** The UV vis absorption spectra of samples.

**Fig. S4.** Plots of (A*hv)^1/2^ versus photon energy (hv) over the catalysts.

**Fig. S5.** The XPS valence band spectra of samples.

**Fig. S6.** Free radical scavenging experiment of samples under illumination.

**Fig. S7.** Photocatalytic degradation of HCHO curve.

**Fig. S8.** Particle sizes and size distributions of WPU dispersions.

**Fig. S9.** Particle sizes and size distributions of samples dispersions.

**Fig. S10.** AFM images of coatings.

**Fig. S11.** The glossiness of samples coatings.

**Fig. S12.** The pencil hardness of samples coatings.

**Fig. S13.** The adhesion of samples coatings.

**Fig. S14.** The adhesion strength of samples coatings.

**Fig. S15.** Hotspot and eye-tracking plots during the eye-tracking session of WTM-X coatings.

**Fig. S16.** Hotspot and eye-tracking plots during the eye-tracking session of WCT-X coatings.

**Fig. S17.** Hotspot and eye-tracking plots during the eye-tracking session of WTN-X coatings.

**Fig. S18.** Hotspot and eye-tracking plots during the eye-tracking session of WCC-X coatings.

**Fig. S19.** Hotspot and Eye-Tracking Trajectory Diagrams.

**Fig. S20.** Accuracy rate for predicting tree species and coatings.

**Fig. S21.** Prediction accuracy of coating ratio.

**Fig. S22.** A Framework for the Intelligent Design of Photocatalytic Composite Coatings.

**Table S1.** Raw data from the left eye of the test subject during the viewing of Red Oak samples.

**Table S2.** Raw data from the left eye of the test subject during the viewing of European Ash samples.

**Table S3.** Raw data from the left eye of the test subject during the viewing of Cherrywood samples.

**Table S4.** Raw data from the left eye of the test subject during the viewing of Sapelli samples.

**Table S5.** Parameters related to the eye tracker viewing process of Red Oak samples

**Table S6.** Parameters related to the eye tracker viewing process of European Ash samples

**Table S7.** Parameters related to the eye tracker viewing process of Cherrywood samples

**Table S8.** Parameters related to the eye tracker viewing process of Sapelli samples

**Table S9.** Relevant parameters during the process of viewing the optimal combination sample using an eye tracker of Red Oak.

**Table S10.** Relevant parameters during the process of viewing the optimal combination sample using an eye tracker of European Ash.

**Table S11.** Relevant parameters during the process of viewing the optimal combination sample using an eye tracker of Cherrywood.

**Table S12.** Relevant parameters during the process of viewing the optimal combination sample using an eye tracker of Sapelli.

**1. Experimental section**

**1.1 Materials**

Ti(OiPr)_4_ (97%), terephthalic acid, 2-amino-1,4-benzenedicarboxylic acid (NH_2_-BDC, AR), methanol (anhydrous 99.9%), N, N-dimethylformamide (anhydrous, 99.8%), acetic acid (anhydrous, 99.9%), TiO_2_ and CuCl_2_ were purchased from Macklin. All reagents and solvents were used as received without further purification.

**1.2 Synthesis of TiO_2_@MIL-125 Catalysts**

Firstly, 500 mg of terephthalic acid was dissolved into 20 mL of DMF:MeOH (9:1 v/v %) solution and mixed, then TiO_2_ nanoparticles were added, followed by 183 µL of Ti(OiPr)_4_. A reactor was heated at 150 °C for 24 h, then washed with DMF and methanol, and finally dried under vacuum at ambient temperature to obtain TiO_2_@MIL-125.

**1.3 Synthesis of TiO_2_@NM-120 Catalysts**

TiO_2_ was dispersed in a mixture of 45 mL N, N-dimethylformamide (DMF) and 5 mL methanol (MeOH). Subsequently, 1.087 g of 2-aminoterephthalic acid, 0.52 ml of Ti(OiPr)_4_, and 120wt% acetic acid was added (120 wt% represents the weight percent of the acetic acid relative to the NH₂-BDC). The solution was maintained at 150 °C for 24 h. Cooling to room temperature, then washed with DMF and methanol. The as-synthesized catalysts were further purification using a hot mixture of ethanol and ethanoic acid. Then the samples were treated using argon plasma at a power of 200 W under low pressure (20 Pa) for a duration of 60 min. Catalysts with varying degrees of structural defects were introduced through the aforementioned treatment and named TiO_2_@NM-120.

**1.4 Synthesis of Cu_0.20_Ti-NM Catalysts**

The Cu_0.20_Ti-NM photocatalysts were synthesized by the photoreduction method. NM was added into 50 mL CuCl_2_ aqueous solution, then ultrasonic treatment for 20 min. Subsequently, the mixed solution was kept stirring under 300 W Mercury lamp illumination at a distance of 15 cm for 1 h. After the reaction, the samples were centrifuged and washed sequentially with deionized water and ethanol. Finally, the Cu_0.20_Ti-NM (0.20 represents the mass fraction of copper ions relative to the catalyst) was obtained after drying at 80°C.

**1.5 Synthesis of Cu-TiO_2_/C Catalysts**

In order to incorporate copper into the MOF crystals, the NM was first dispersed in aqueous CuCl_2_ solution and sonicated for 1 hour. In situ nitrogen/carbon co-doped Cu-TiO_2_/C nanocomposites were prepared by one-step direct carbonization of template NM (Cu) at 600 °C for 2 h with a heating rate of 2 °C min-1 under N_2_ flow.

**1.6 Synthesis of NM Catalysts**

1.087 g of 2-aminoterephthalic acid, 0.52 ml of Ti (OiPr)_4_, and 5 ml of methanol (MeOH) were dissolved in 45 ml of N, N-dimethylformamide (DMF). Then, 120wt% acetic acid was added (120 wt% represents the weight percent of the acetic acid relative to the NH₂-BDC). The solution was transferred to a reaction vessel and maintained at 150 °C for 24 hours. After cooling to room temperature, it was washed with DMF and methanol. Further purification was conducted using a hot mixture of ethanol and acetic acid. Under low pressure (20 Pa), the above sample was treated with a 200 W argon plasma for 60 minutes, obtained NM (Defective NH_2_-MIL-125). NH_2_-MIL-125 was synthesized without acetic acid and plasma treatment.

**1.7 Synthesis of Coating**

TiO_2_@MIL-125，TiO_2_@NM-120, Cu_0.20_Ti-NM and Cu- TiO_2_/C were dispersed in ethanol (EtOH) solution and sonicated, respectively. Then, waterborne polyurethane (WPU) was added to the mixture and sonicated to obtain a stable suspension. The obtained suspension was sprayed onto the wood substrate (10cm*10cm*1cm) (Cherrywood, Red Oak, Sapelli and European Ash) to form a WTM-X, WTN-X, WCT-X and WCC-X composite coating at a pressure between 60 and 90 psi with a spraying distance of 10 cm. X represents the mass fraction of catalysts relative to the waterborne polyurethane (in five mass ratios 1wt%, 2wt%, 3wt%, 4wt%, and 5wt%).

**2. Characterization**

The crystalline structure of the sample was analyzed using X-ray diffraction (XRD) (X, Pert3 Powedr, Netherlands). Analysis of the morphological characteristics of the synthesized sample was performed using a scanning electron microscope (SEM, Gemini SEM 300, ZEISS). Assessment of the chemical structures was carried out using Fourier Transform Infrared (FTIR) spectroscopy (Bruker Tensor II), with a spectral resolution of 4 cm^-^¹, 32 scans, and a range of 500-4000 cm^-^¹. The UV-Vis diffuse reflectance spectra were obtained using a Cary 100 spectrophotometer (Agilent Technologies Inc., USA).

According to ASTM Standard D523, the surface gloss of wood coating was measured using a WGG-60 gloss meter (Hangzhou Qiwei Instrument Co., Ltd., Hangzhou, China) at an incident angle of 60° [1]. As per ASTM Standard D3363-20, the hardness of the wood surface coatings was evaluated using a QHQ-A commercial pencil hardness tester (Tianjin Jingke Material Testing Instruments Co., Ltd., Tianjin, China), with pencil hardness grades ranging from 2B to 5H [2]. Additionally, the adhesion strength of the wood coatings was tested in accordance with ASTM D4541 [3] via a pull-off test. A 20 mm diameter dolly was glued onto the coated wood surface and pulled until detachment occurred, with the maximum adhesion strength at failure recorded in megapascals (MPa).

**2.1 Gaseous formaldehyde photodegradation**

The photocatalytic degradation of formaldehyde (HCHO) was conducted in a quartz reactor (Fig. S1). A circulating water system was employed to minimize environmental variables during the experiment. A 300 W xenon lamp with a wavelength range of 200-1100 nm and an incident light intensity of approximately 800 mW/cm² was used as the light source. The initial formaldehyde concentration was 5 ppm. During the experiment, real-time HCHO concentrations were measured using a formaldehyde analyzer (Formaldemeter HTV-M, PPM Technology). The catalysts were placed in a dark environment under an HCHO atmosphere until adsorption-desorption equilibrium was reached, defining the initial formaldehyde concentration as C_0_. The real-time formaldehyde concentration at any given time was recorded as Cₜ. The formaldehyde degradation efficiency (η_HCHO_) was calculated Eq. (1)[4]:

η_HCHO_ (%) = (C_0_-C_t_) C_0_×100 (1)

where C₀ is the initial formaldehyde concentration and Cₜ is the concentration at time t. To ensure data reliability, each photocatalytic degradation experiment was repeated three times.

**2.2 Photoelectrochemical measurements**

A CHI-760E electrochemical workstation (Shanghai Chenhua Instrument Co., Ltd., China) was used to conduct photoelectrochemical measurements within a standard three-electrode configuration. Electron paramagnetic resonance (EPR) spectroscopy (Bruker EMXplus-6/1, Germany) was employed to detect and confirm the presence and identity of radical species involved in the photocatalytic processes.

**2.3 Free radical scavenging experiments**

A 5 mM solution of tert-butanol (TBA, a hydroxyl radical scavenger) and 5 mM solution of benzoquinone (BQ, a superoxide radical scavenger) were individually added to 5 mL of water and dispersed uniformly. Subsequently, the catalyst was introduced into the aforementioned solution, followed by ultrasonication for 10 minutes. The mixture was then poured into a petri dish and dried at 60 °C. The composite material was subsequently placed into the reactor for radical scavenging experiments. The reported data represent the mean values of three independent measurements, with standard error calculated accordingly.

**2.4 Color Testing of Coated Wood Surfaces**

A spectrophotometer (DS-200) was used to measure the color of wood samples before and after coating. During the testing process, the center point of each wood sample was selected, and each sample was tested three times to calculate the average value. The data was then analyzed and compared using the CIE (1976) L*, a*, b* colorimetric space color system. The CIE (1976) L*, a*, b* colorimetric space consists of three coordinate axes: the lightness index L*, the a* chromaticity index on the red-green axis, and the b* chromaticity index on the yellow-blue axis.

**2.5 Eye-Tracking Experiment on Attention to Coating Colors**

Digital photography was used to capture and record coating information for color eye-tracking experiments. Coatings of the same wood species treated with different catalyst ratios were grouped together for viewing tests, with six different ratios designated as six regions of interest.

The Wearable Eye-Tracking System “Dikablis Glasses B 17302” from the Germany company ERGONEERS was used. Tracking accuracy: 0.3^°^-0.5^°^ Sampling frequency: 50 Hz. Built-in QR code AOI technology for automatic analysis of areas of interest. Along with a 15.6-inch square display with a resolution of 3840 × 2160 pixels and a sampling frequency of 60 Hz. The experiment was conducted in a well-soundproofed room free from environmental interference. All participants had no color blindness or color vision deficiency and possessed good visual acuity. They had no physical impairments that could affect visual perception. The study primarily investigated which coating colors the subjects focused on more among different coating colors. During the experiment, the D-Lab software (eye tracker data acquisition and analysis software) was launched, color images were imported into the experimental computer, and the subjects were seated 50-80 cm in front of the computer. A total of 20 sets of images were used, with each set displayed for 15 seconds (a duration short enough to prevent the images from becoming blurred, yet long enough to avoid inducing visual fatigue). A preliminary test was conducted before the main experiment to verify the accuracy of the participants’ gaze patterns. Equipment calibration was performed for each participant after their test[5]. All human participants involved in this eye-tracking experiment provided written informed consent prior to data collection. Participants were allowed to stop the test voluntarily at any moment.

**2.6 Machine Learning Computational Details**

The experiment aimed to establish a predictive relationship between material visual features and human visual attention. Each material sample was assigned a single attention label derived from eye-tracking experiments, where attention metrics such as average fixation duration and fixation density were aggregated across 20 participants. Based on these aggregated metrics, attention labels were discretized into three levels (low, medium, and high).

A gradient-boosted decision tree model (XGBoost classifier) was employed to predict attention levels from material and visual features. The classification task was formulated at the material-sample level, with each sample represented by a single feature vector and a corresponding attention label.

Inverse material design was performed using a framework that combines forward surrogate modeling with Bayesian optimization. The forward mapping between material parameters and output properties was modeled using Gaussian process regression (GPR)[6]. Given the limited number of experimental samples, independent Gaussian process models were trained for each output variable (Lab, and formaldehyde degradation efficiency). The predictive means of these surrogate models were used to construct an objective function for inverse design. The objective function quantified the discrepancy between surrogate-predicted outputs and specified target outputs by jointly constraining deviations in perceptual color parameters and functional performance through a weighted sum of squared errors.

The inverse design task was formulated as an optimization problem over a mixed discrete-continuous design space[7]. Bayesian optimization problem over a iteratively search for candidate material configurations under a fixed evaluation budget[8]. A lower confidence bound acquisition function was used to guide the search by balancing exploration and exploitation. Each inverse search was initialized with a set of randomly sampled configurations, followed by a fixed number of Bayesian optimization iterations. To account for the potential non-uniqueness of the inverse problem, the optimization procedure retained the Top-K candidate configurations with the lowest objective values across the entire search history, rather than returning a single optimal solution.

$$\min_{x} \mathcal{L(}x)=\left\| LAB(x)-\mathrm{LAB}^{*} \right\|_{2}^{2}+\left\| \mathrm{HCO}(x)-\mathrm{HCO}^{*} \right\|_{2}^{2}$$

To evaluate generalization under unseen composition conditions, a leave-one-ratio-out strategy was adopted. In each validation round, all samples associated with one coating ratio were excluded from training of the forward surrogate models, which were trained using data from the remaining ratios. Inverse design was then performed using only the target outputs associated with the excluded ratio, ensuring that the inverse search was conducted under held-out ratio conditions.

**
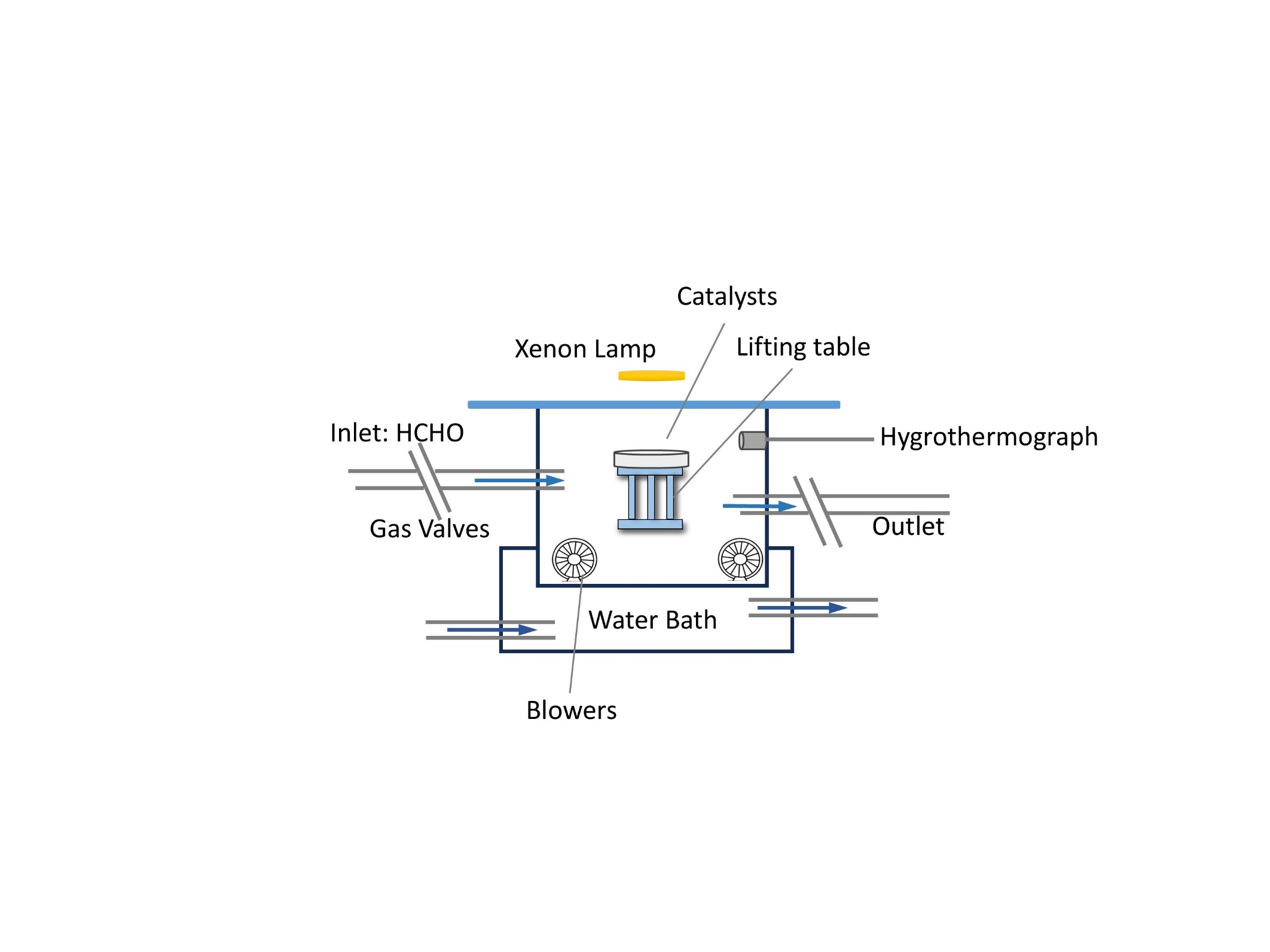
**

**Fig. S1.** The schematic illustration of the photocatalytic test.


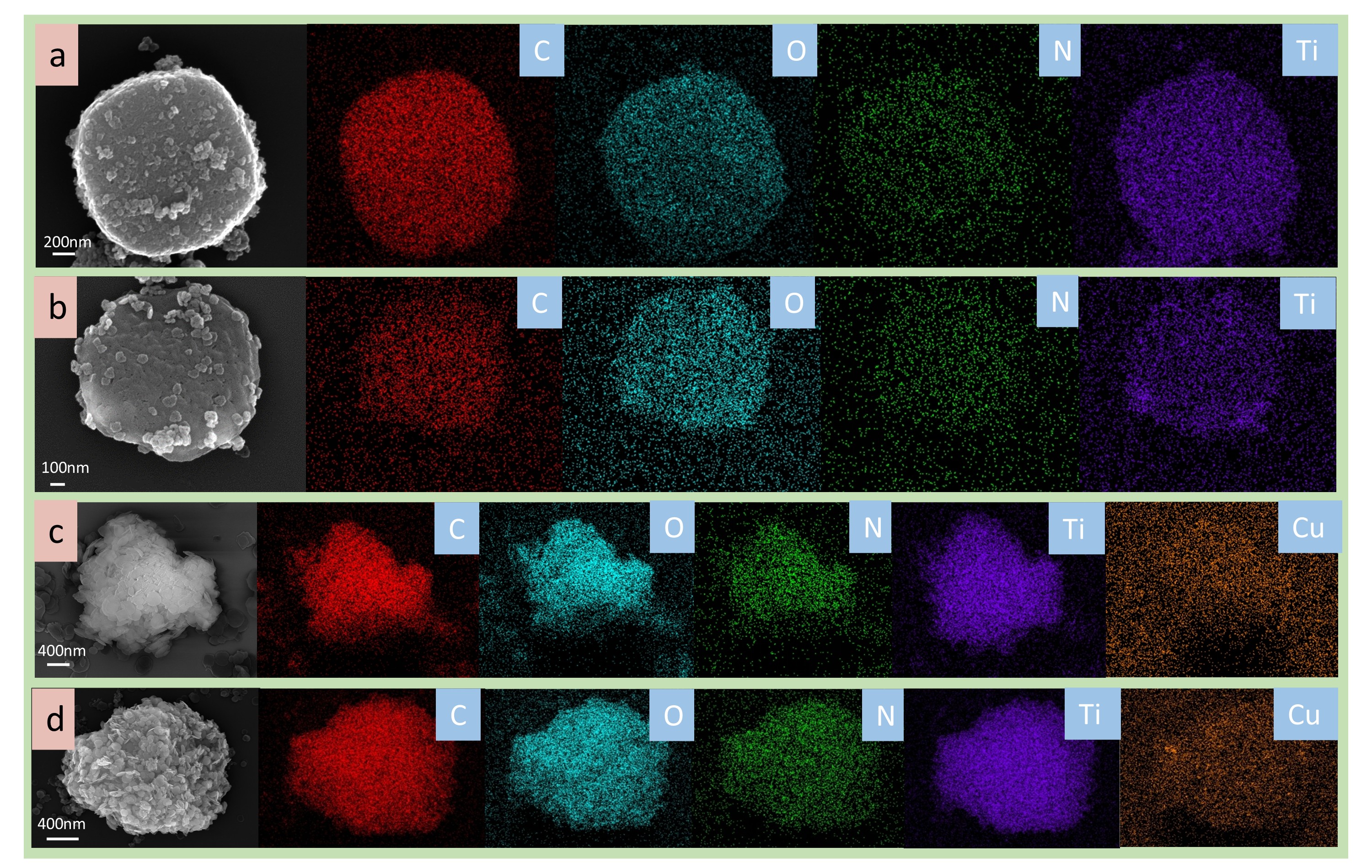


**Fig. S2.** The EDS element mappings of (a) TiO_2_@MIL-125; (b) TiO_2_@NM-120; (c) Cu_0.20_Ti-NM; (d) Cu-TiO_2_/C.


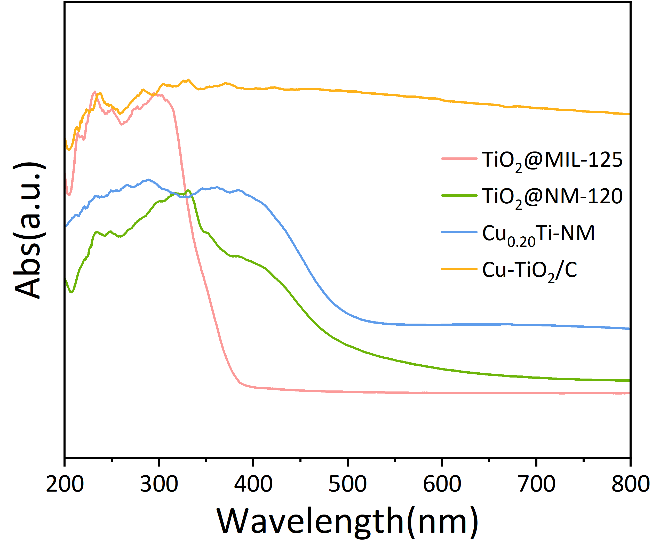


**Fig. S3.** The UV vis absorption spectra of samples.

**
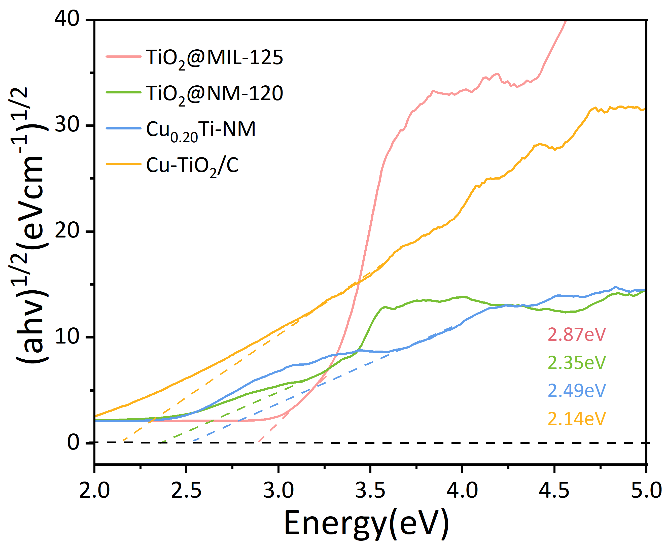
**

**Fig. S4.** Plots of (A**hv*)^1/2^ versus photon energy (*hv*) over the catalysts.

**
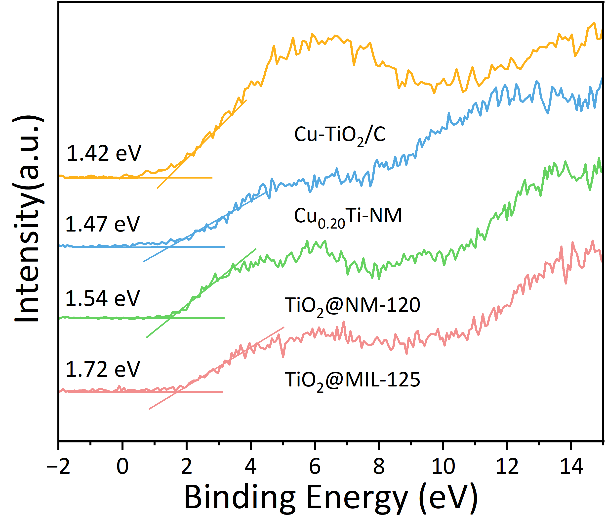
**

**Fig. S5.** The XPS valence band spectra of samples.

**
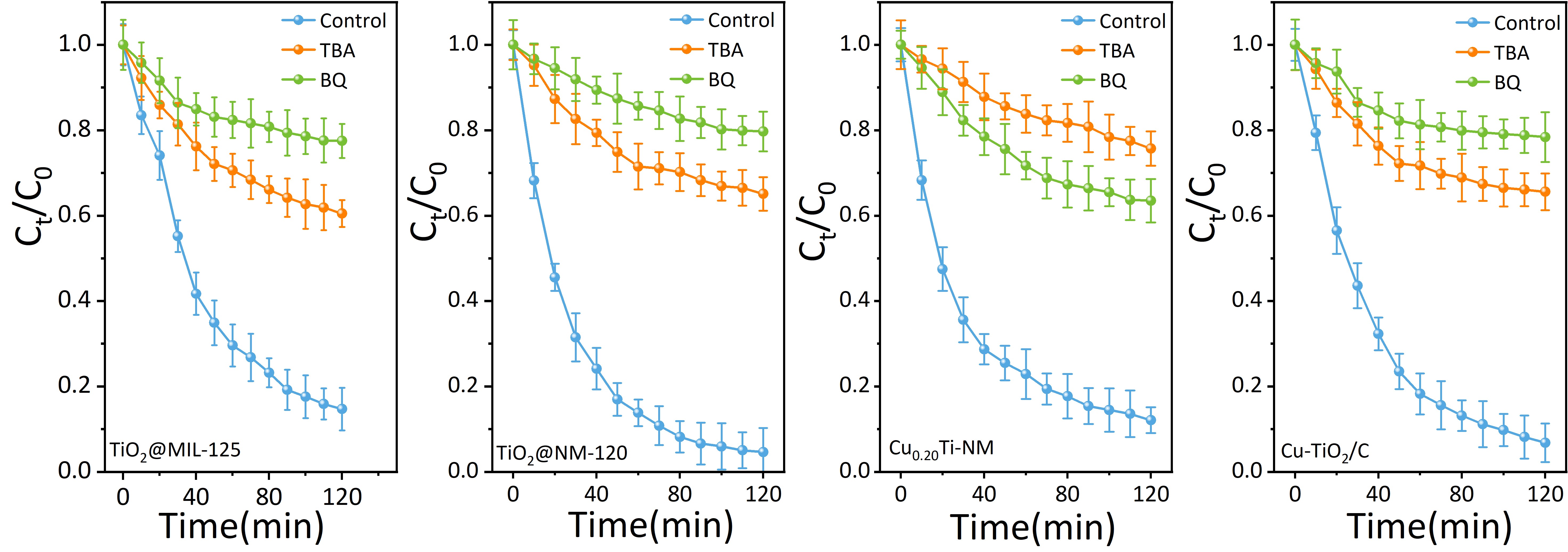
**

**Fig. S6.** Free radical scavenging experiment of samples under illumination.

**
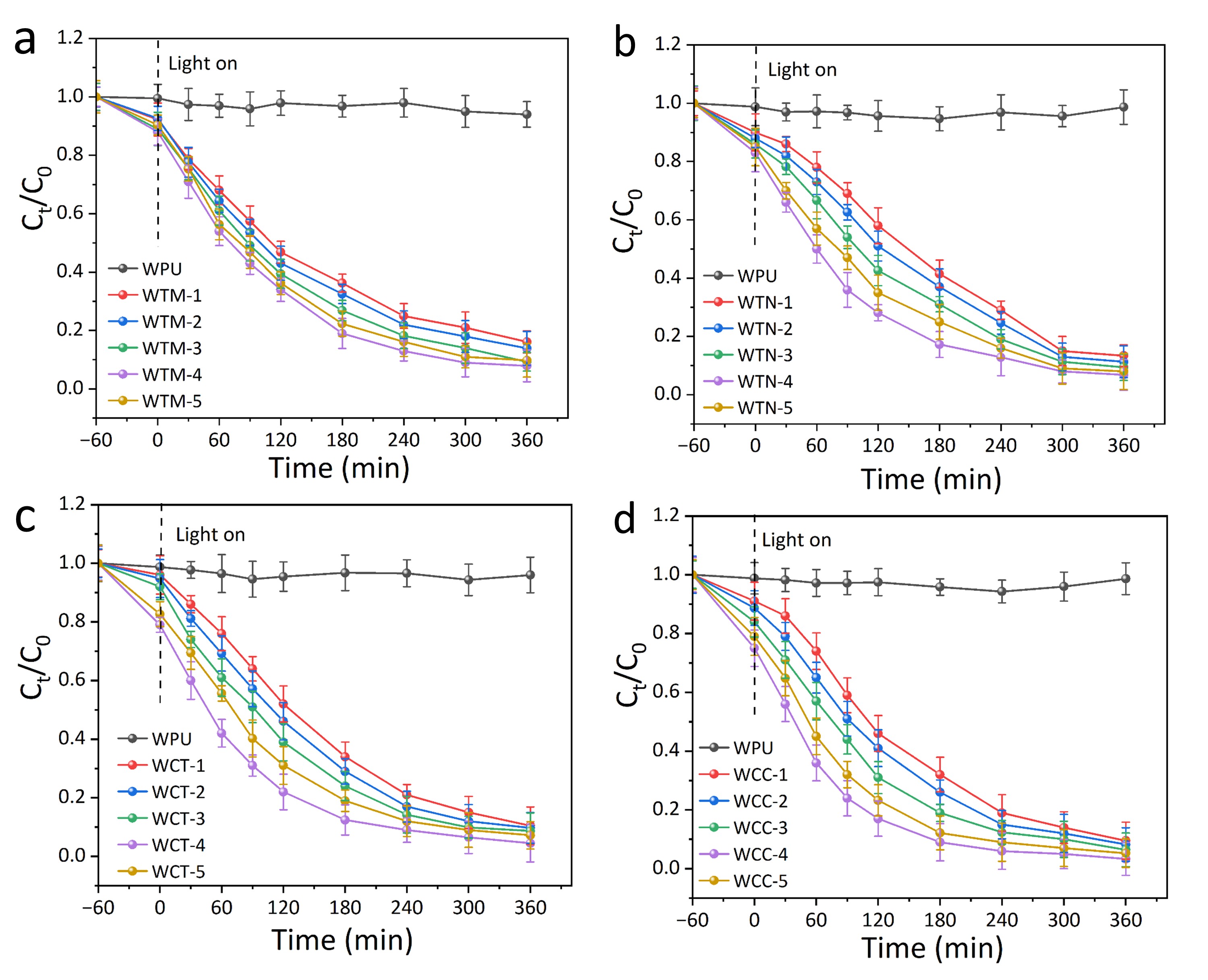
**

**Fig. S7.** Photocatalytic degradation of HCHO curve.


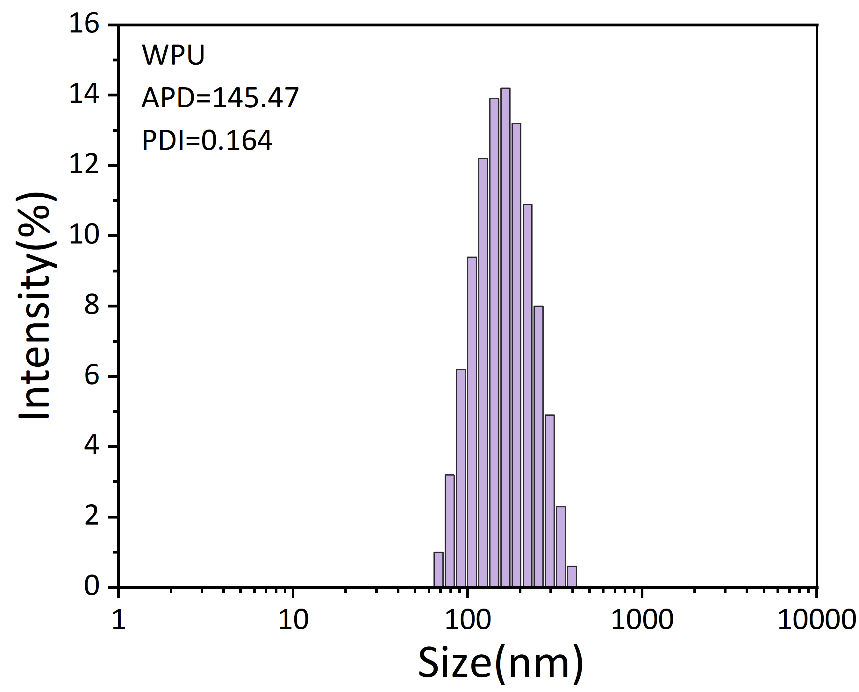


**Fig. S8.** Particle sizes and size distributions of WPU dispersions.


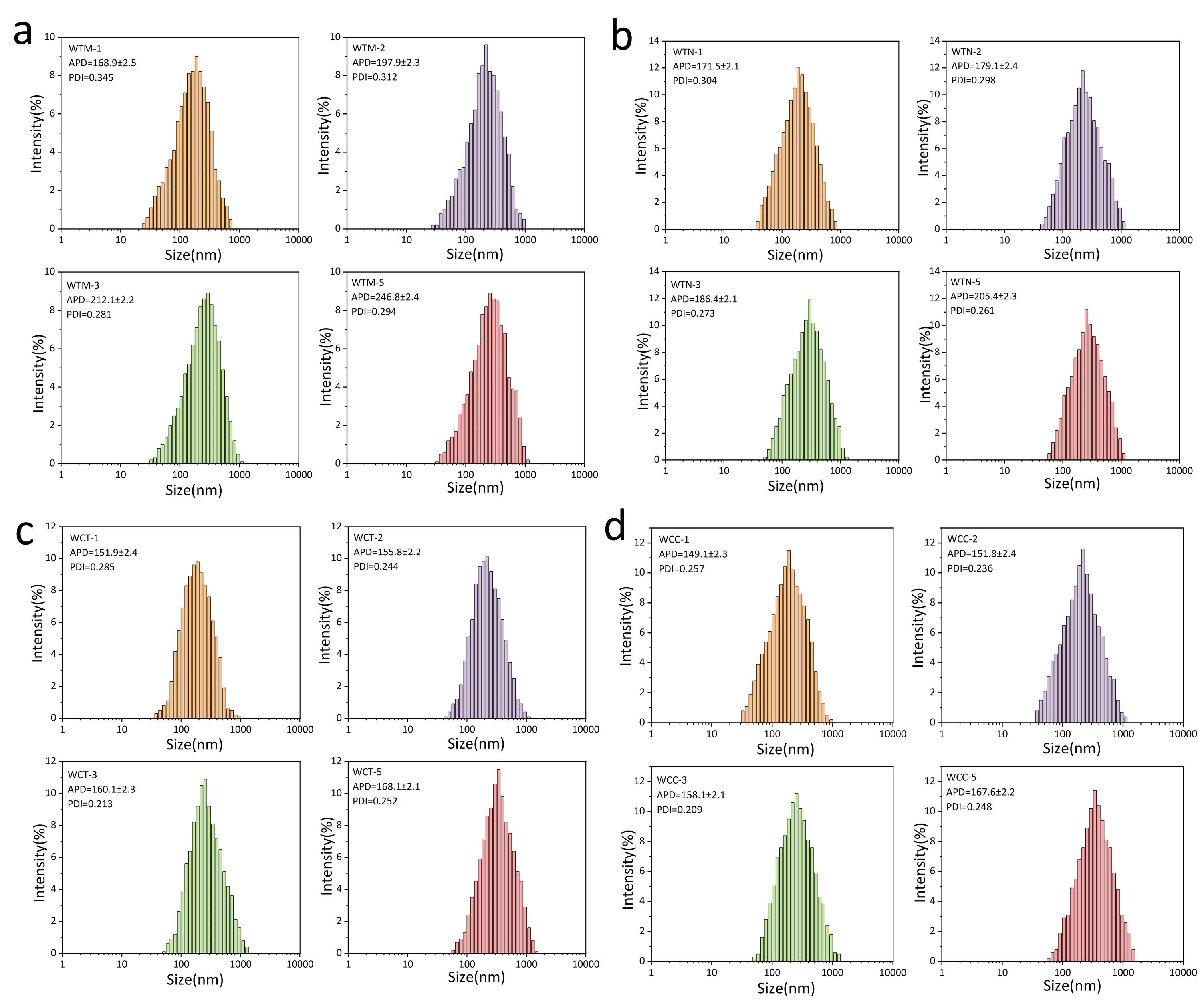


**Fig. S9.** Particle sizes and size distributions of samples dispersions.


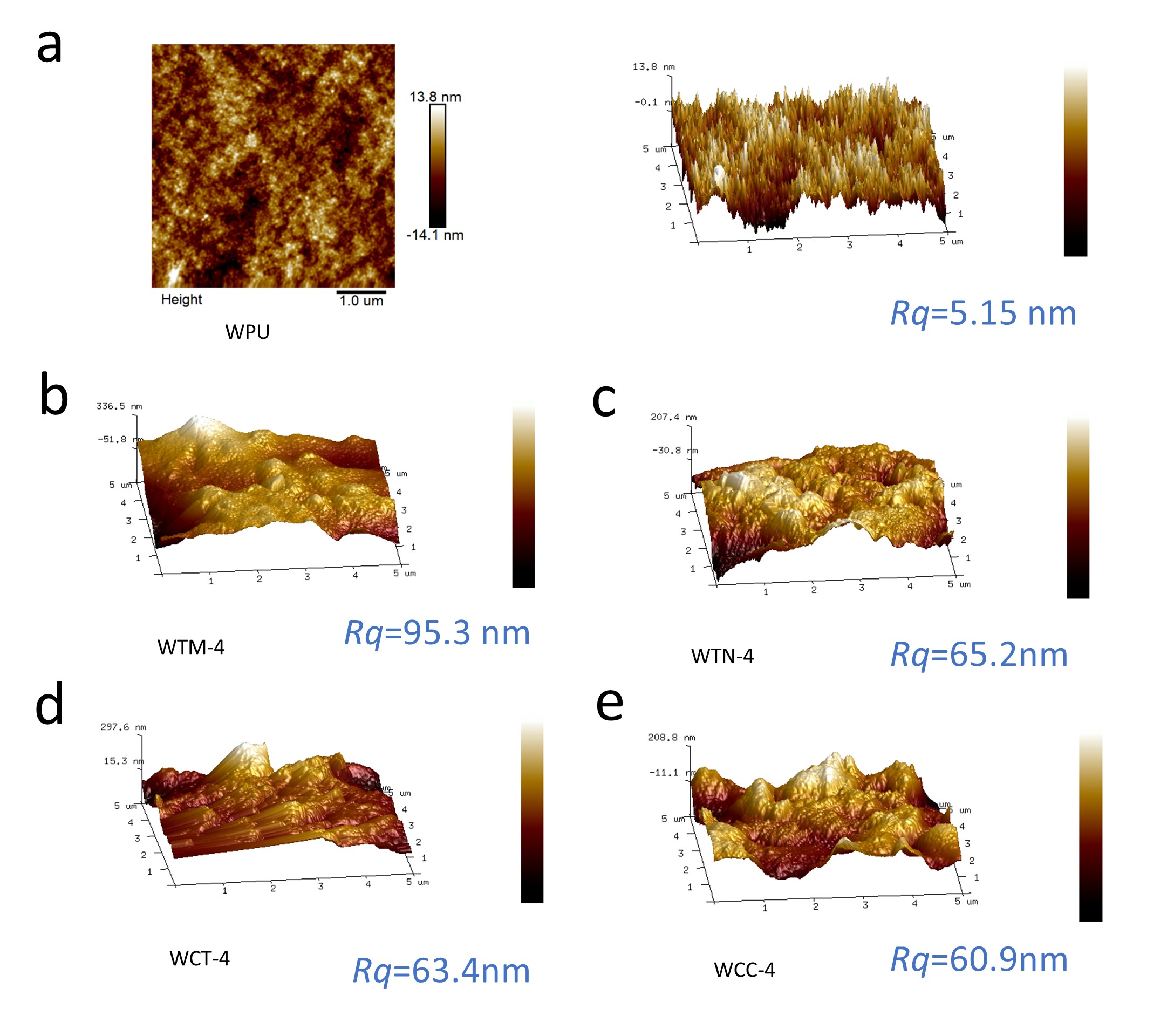


**Fig. S10.** AFM images of (a) WPU coatings; (b) WTM-4 coatings; (c) WTN-4 coatings; (d) WCT-4 coatings; (e) WCC-4 coatings.


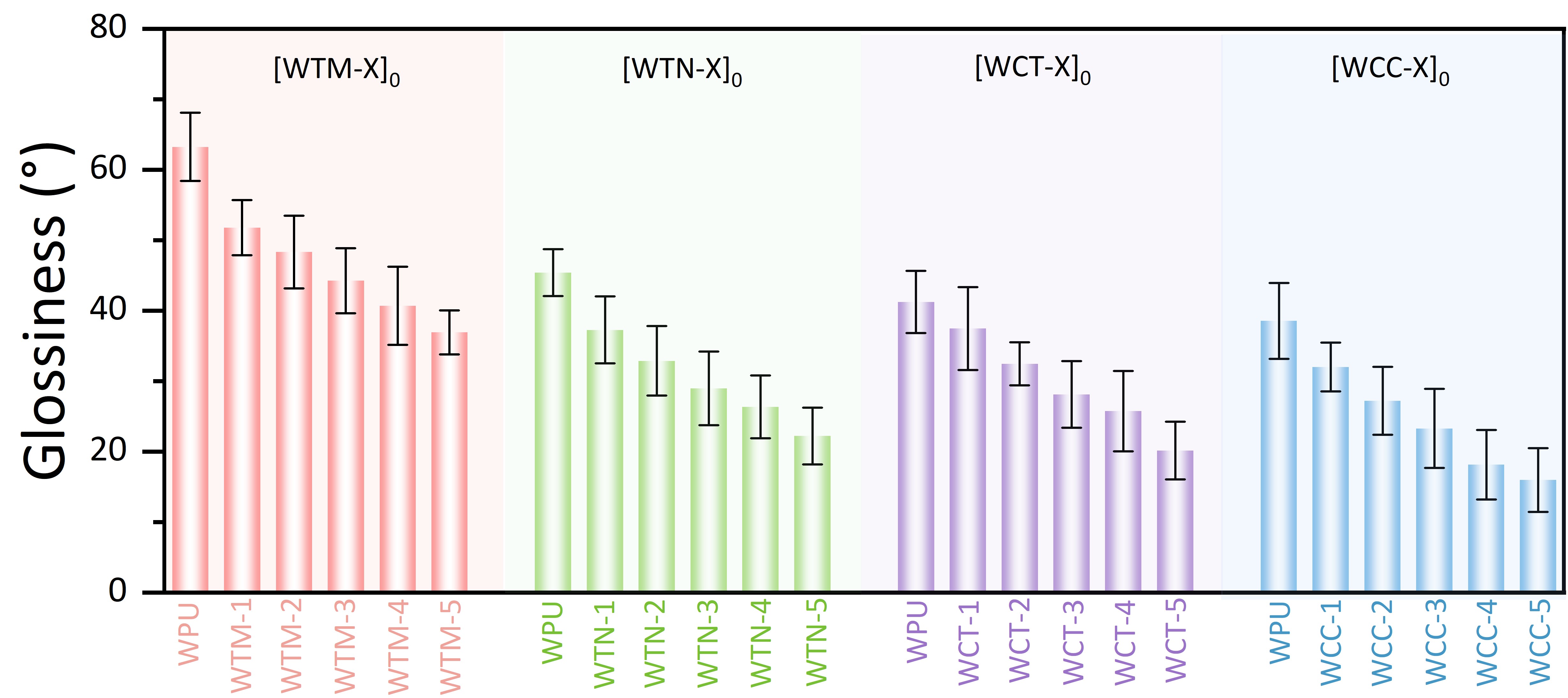


**Fig. S11.** The glossiness of samples coatings.

**
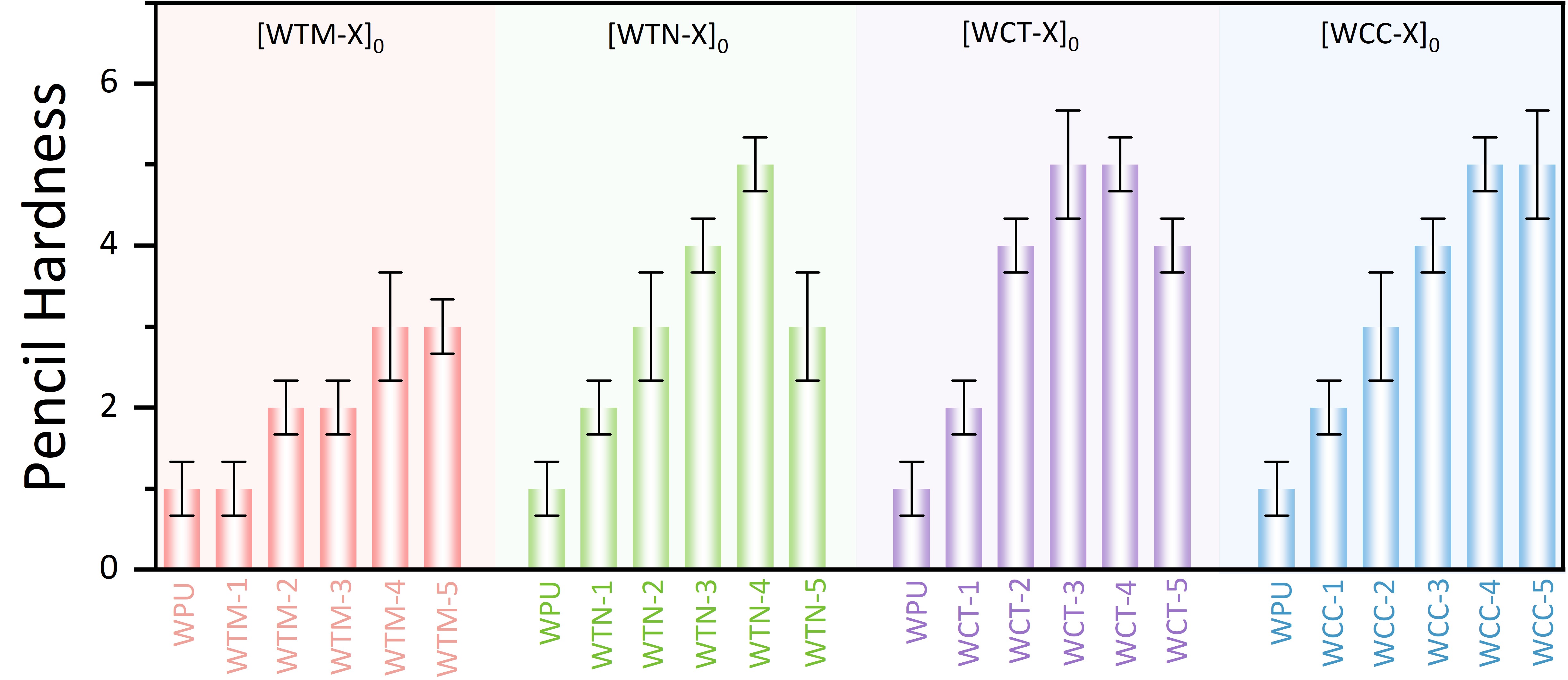
**

**Fig. S12.** The pencil hardness of samples coatings.


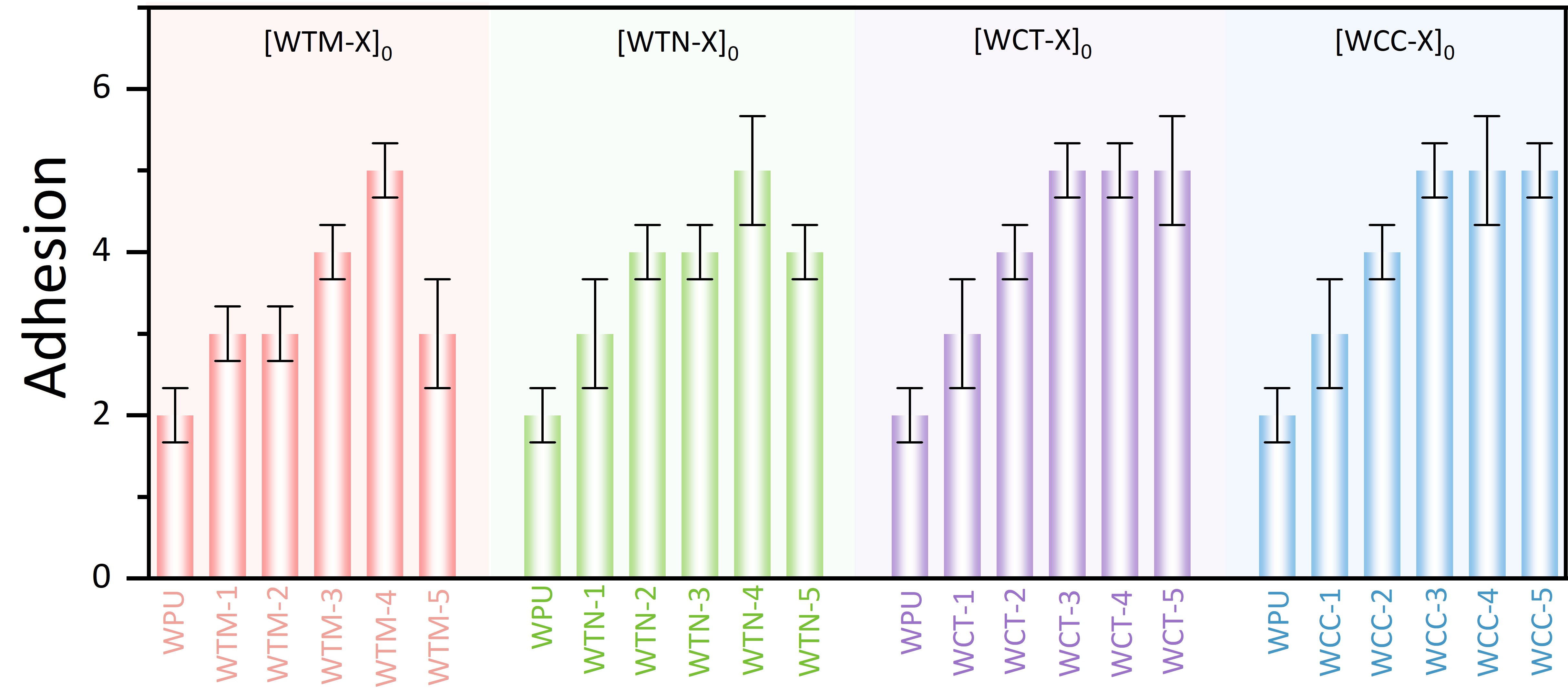


**Fig. S13.** The adhesion of samples coatings.


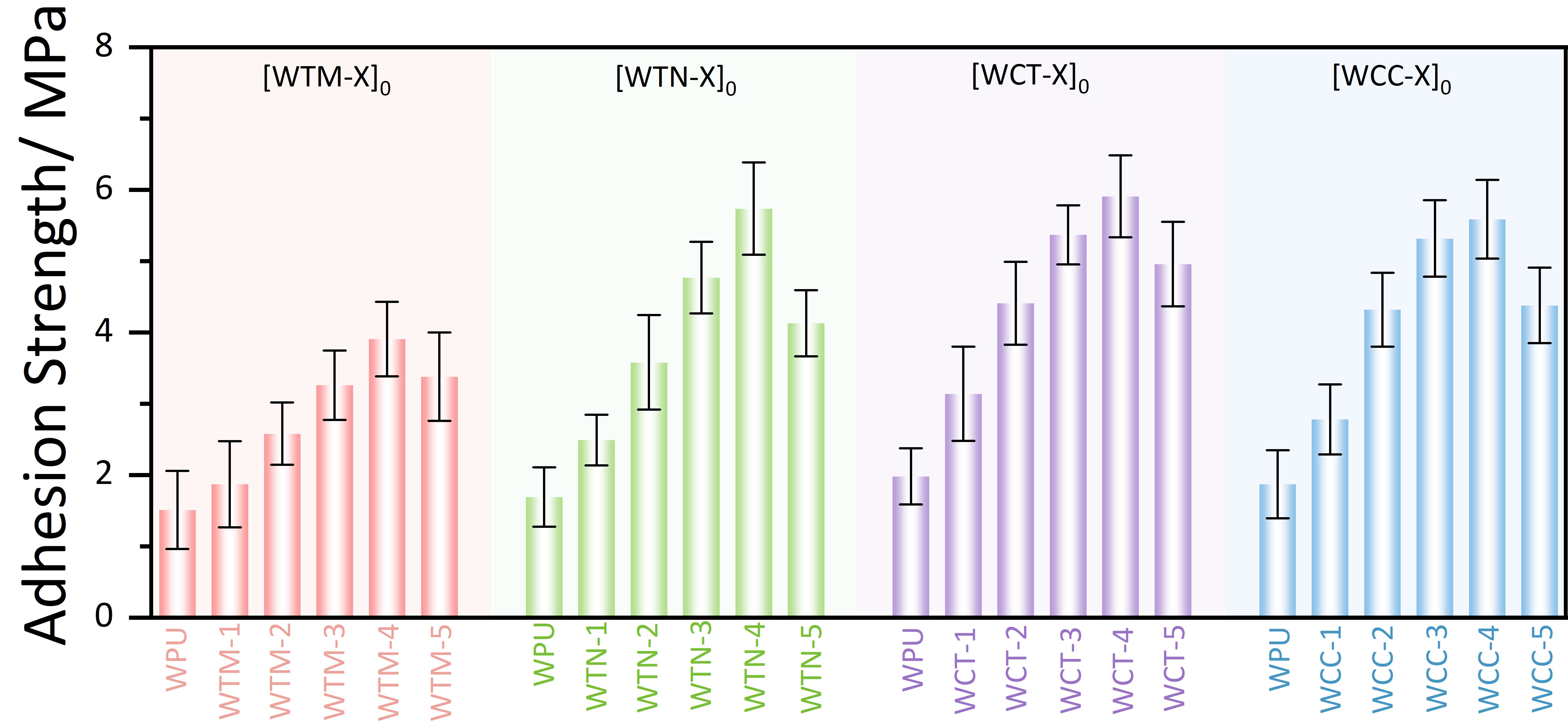


**Fig. S14.** The adhesion strength of samples coatings.


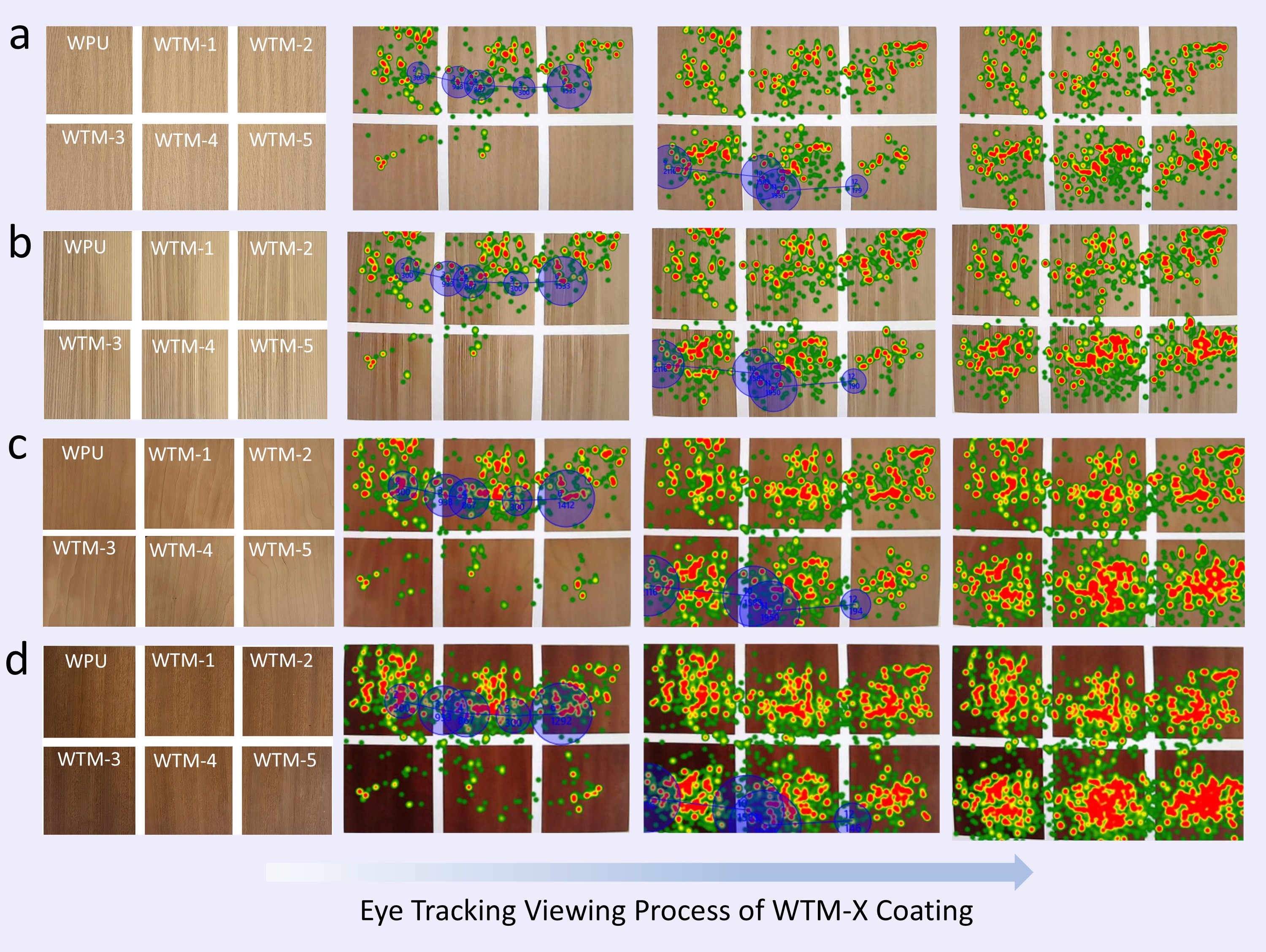


**Fig. S15.** Hotspot and eye-tracking plots during the eye-tracking session of WTM-X coatings for different tree species (a) Red Oak; (b) European Ash; (c) Cherrywood; (d) Sapelli.

**
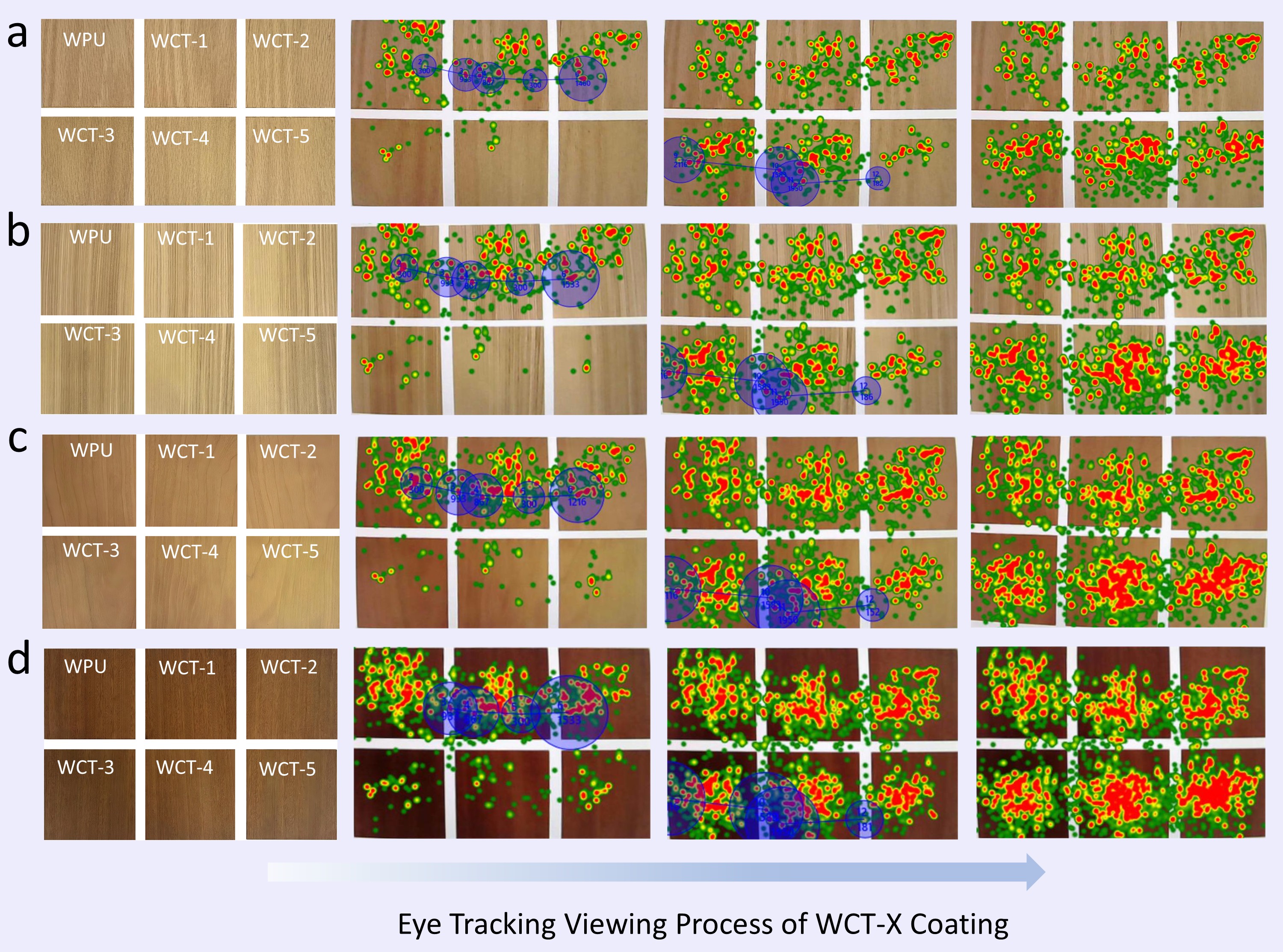
**

**Fig. S16.** Hotspot and eye-tracking plots during the eye-tracking session of WCT-X coatings for different tree species (a) Red Oak; (b) European Ash; (c) Cherrywood; (d) Sapelli.


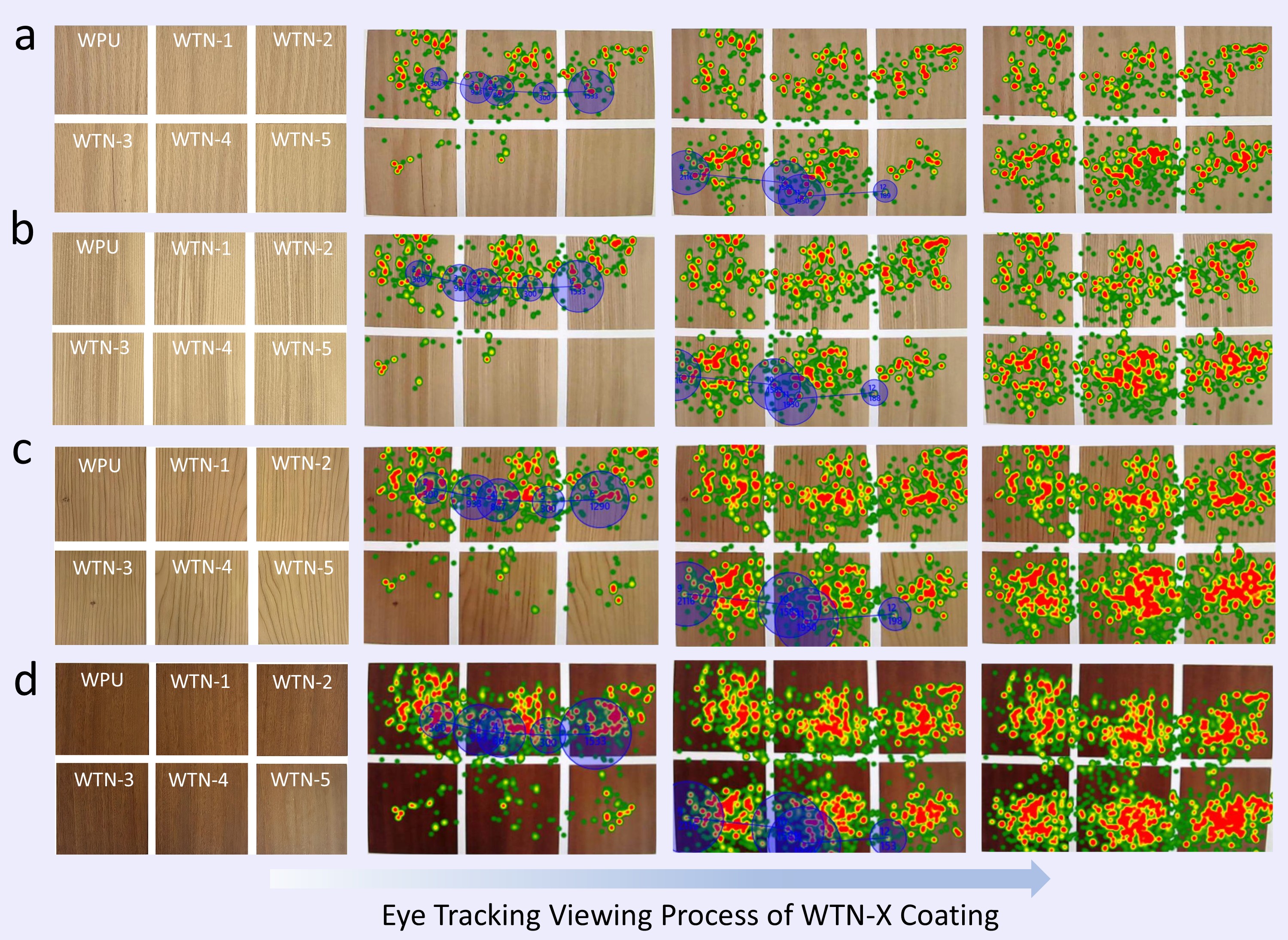


**Fig. S17.** Hotspot and eye-tracking plots during the eye-tracking session of WTN-X coatings for different tree species (a) Red Oak; (b) European Ash; (c) Cherrywood; (d) Sapelli.


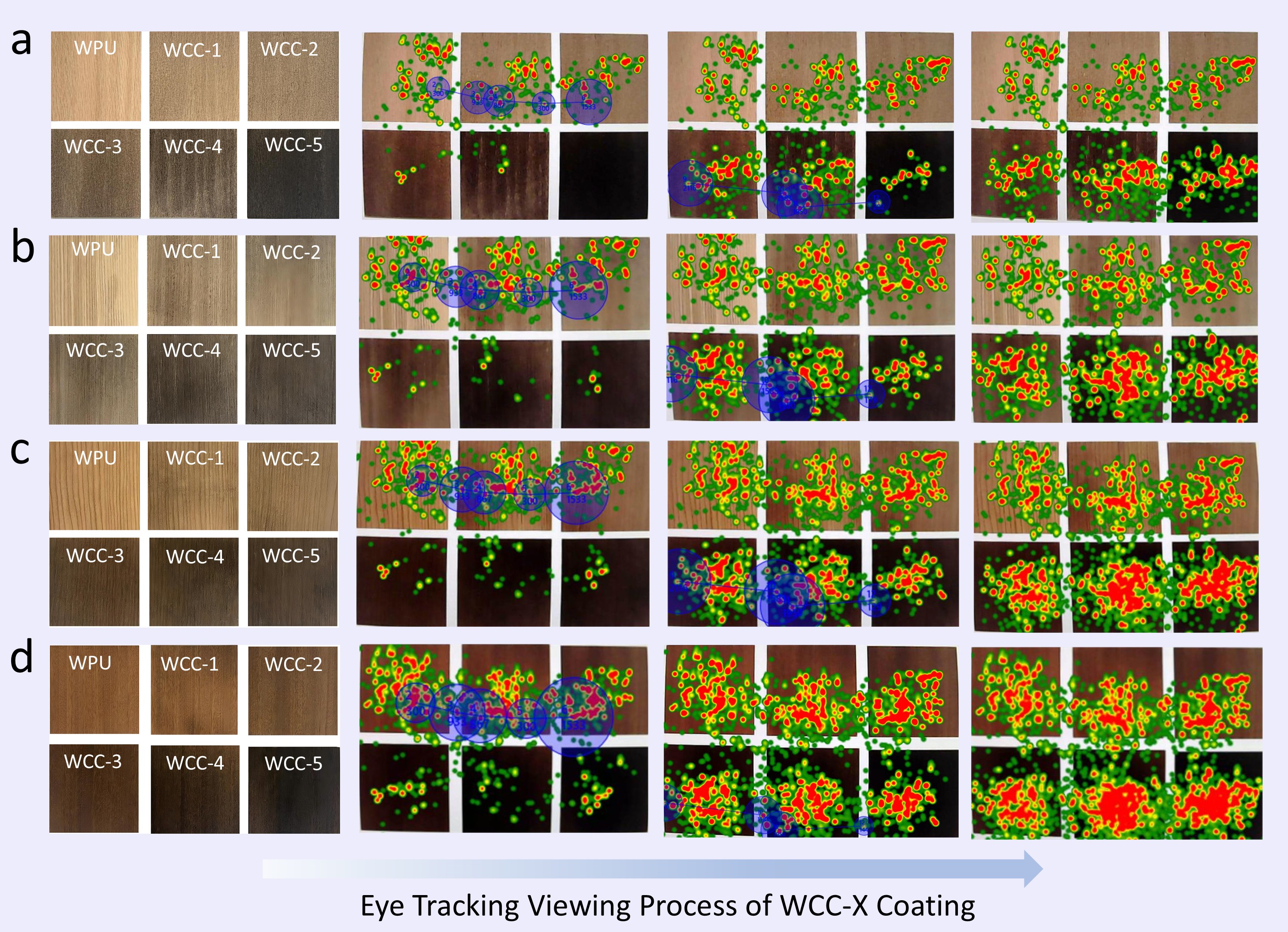


**Fig. S18.** Hotspot and eye-tracking plots during the eye-tracking session of WCC-X coatings for different tree species (a) Red Oak; (b) European Ash; (c) Cherrywood; (d) Sapelli.

**
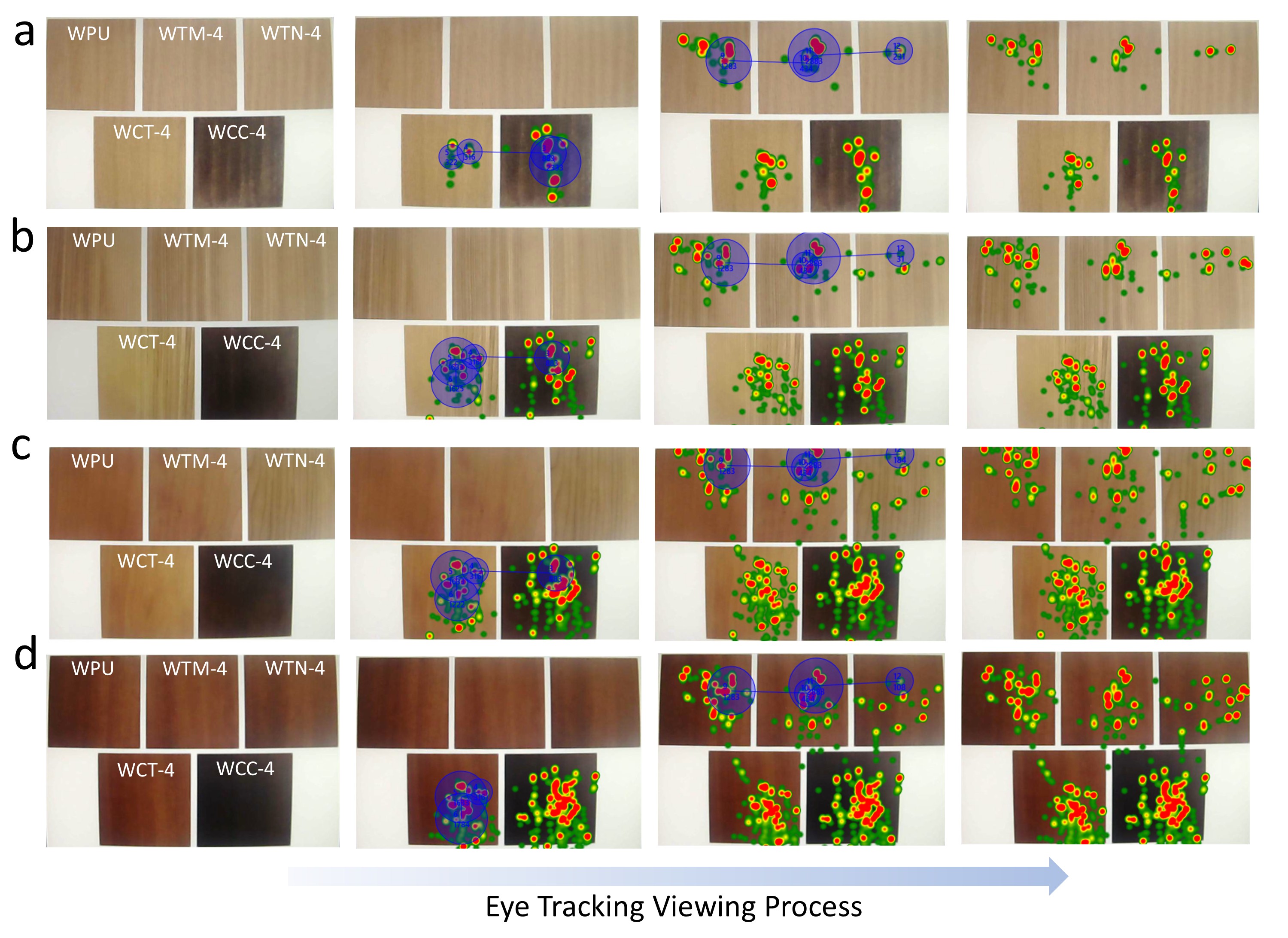
**

**Fig. S19.** Hotspot and Eye-Tracking Trajectory Diagrams for WPU, WTM-4, WTN-4, WCT-4, and WCC-4 Across Different Tree Species in Eye-Tracking Experiments: (a) Red Oak; (b) European Ash; (c) Cherrywood; (d) Sapelli.


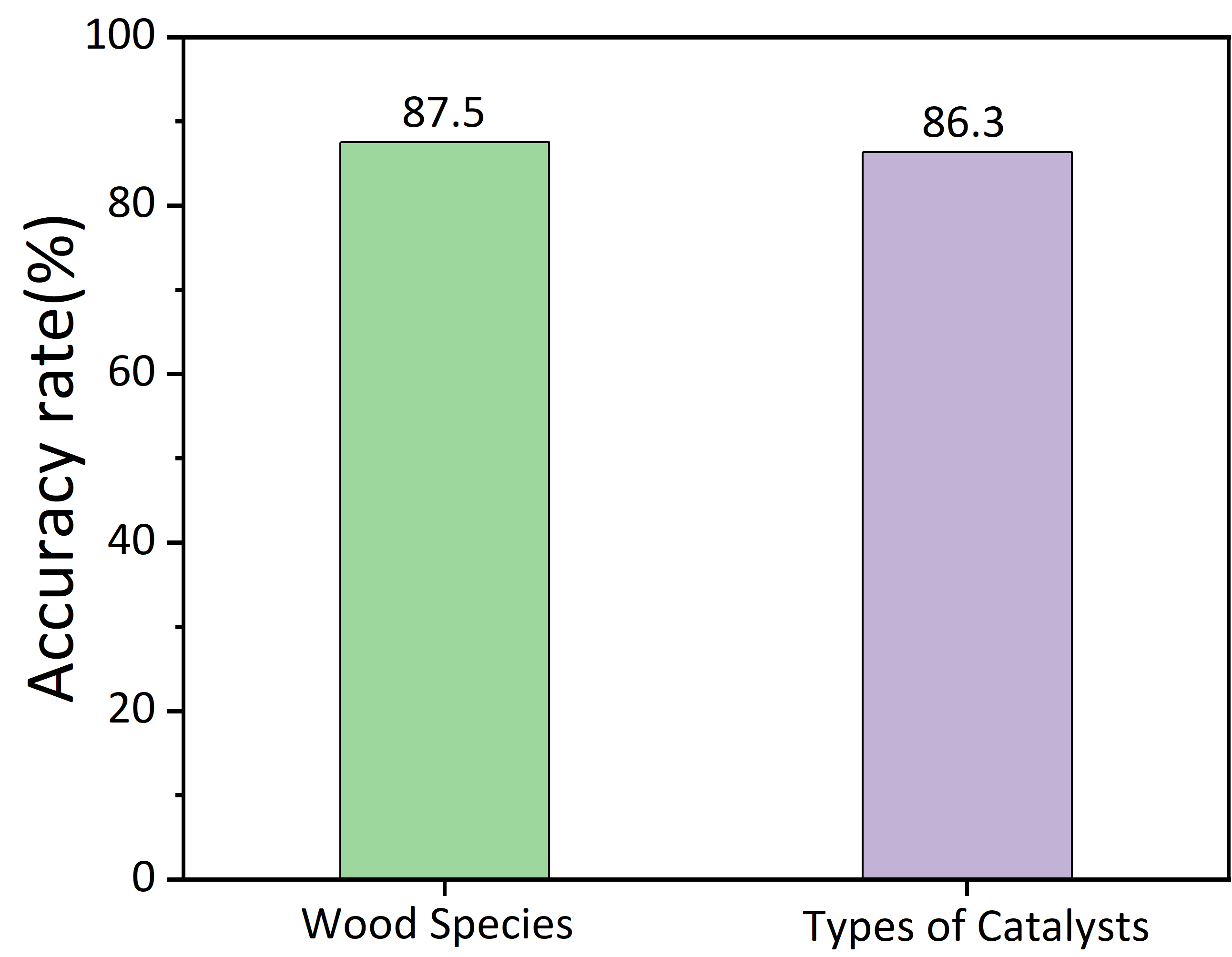


**Fig. S20.** Accuracy rate for predicting tree species and coatings.


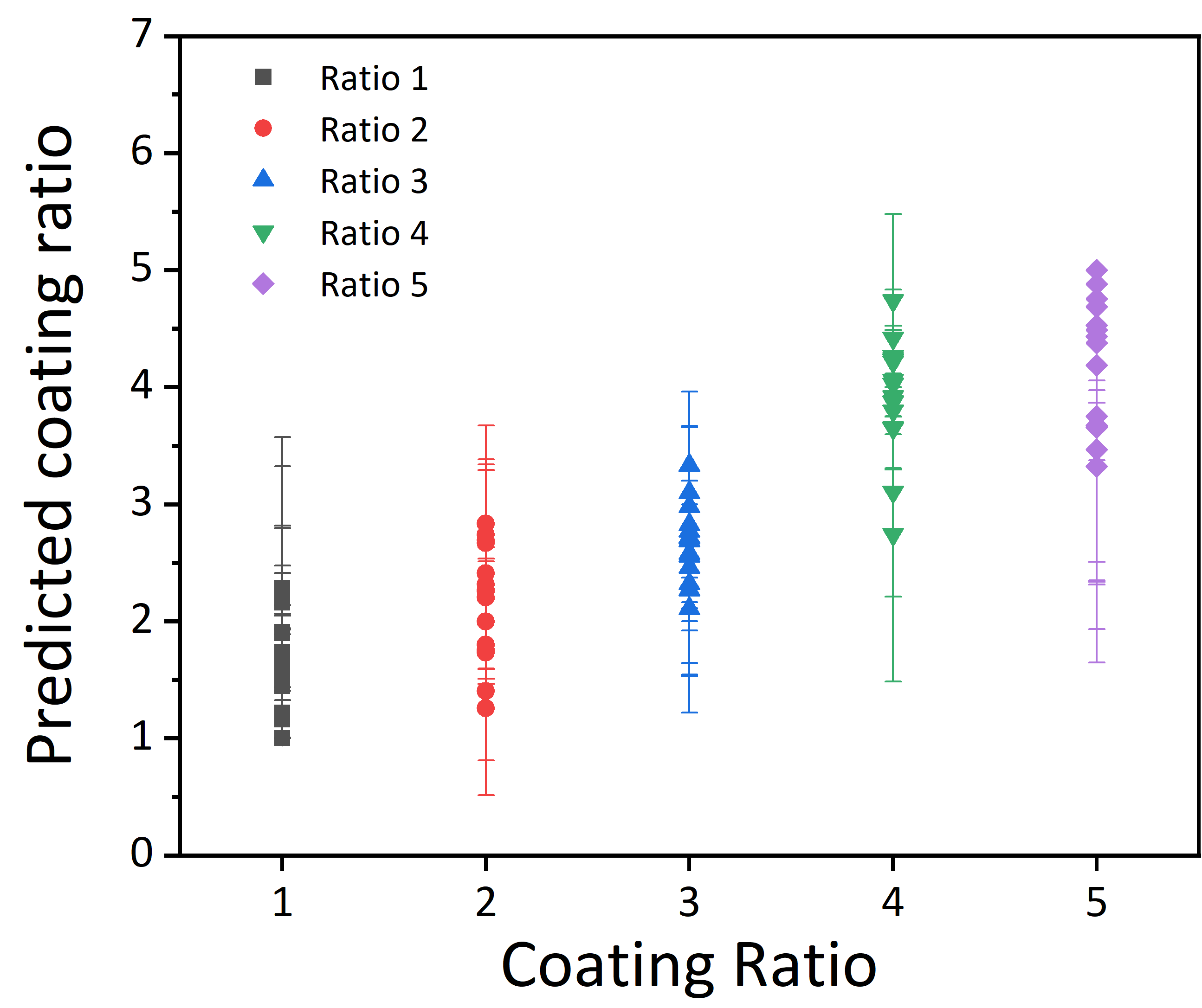


**Fig. S21.** Prediction accuracy of coating ratio.


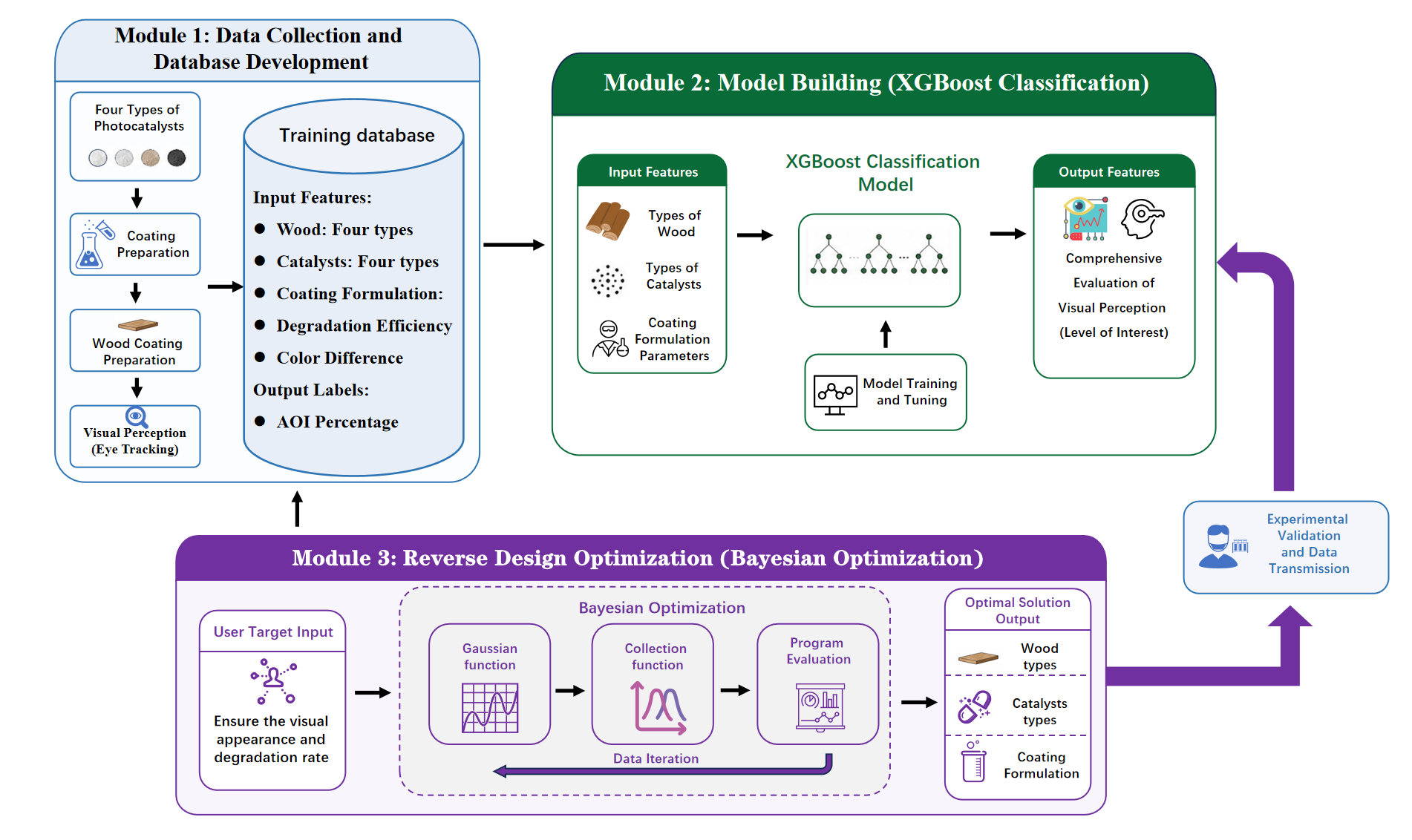


**Fig. S22.** A Framework for the Intelligent Design of Photocatalytic Composite Coatings.

**Table S1.** Raw data from the left eye of the test subject during the viewing of Red Oak samples.

| Samples | Left Eye  Pupil Area  （mm^2^） | Left Eye Pupil Height  （mm） | Left Eye  Pupil Width  （mm） | Samples | Left Eye  Pupil Area  （mm^2^） | Left Eye Pupil Height  （mm） | Left Eye  Pupil Width  （mm） |
| --- | --- | --- | --- | --- | --- | --- | --- |
| WPU | 613.069 | 30.069 | 25.973 | WPU | 626.474 | 30.838 | 25.866 |
| WTM-1 | 647.250 | 29.729 | 27.721 | WTN-1 | 643.909 | 30.074 | 27.261 |
| WTM-2 | 703.423 | 32.055 | 27.94 | WTN-2 | 662.365 | 31.299 | 26.945 |
| WTM-3 | 733.107 | 31.794 | 29.359 | WTN-3 | 713.244 | 31.180 | 29.126 |
| WTM-4 | 741.662 | 31.033 | 30.429 | WTN-4 | 736.364 | 31.030 | 30.215 |
| WTM-5 | 671.209 | 30.503 | 28.017 | WTN-5 | 658.323 | 29.306 | 28.601 |
| WPU | 638.194 | 30.202 | 26.904 | WPU | 542.07 | 28.590 | 24.141 |
| WCT-1 | 675.964 | 30.463 | 28.253 | WCC-1 | 608.998 | 30.391 | 25.514 |
| WCT-2 | 706.352 | 31.472 | 28.576 | WCC-2 | 627.184 | 30.099 | 26.531 |
| WCT-3 | 726.005 | 31.854 | 29.019 | WCC-3 | 650.038 | 30.561 | 27.082 |
| WCT-4 | 742.133 | 32.348 | 29.211 | WCC-4 | 706.817 | 31.691 | 28.397 |
| WCT-5 | 651.019 | 29.51 | 28.088 | WCC-5 | 631.862 | 30.065 | 26.759 |

**Table S2.** Raw data from the left eye of the test subject during the viewing of European Ash samples.

| Samples | Left Eye  Pupil Area  （mm^2^） | Left Eye Pupil Height  （mm） | Left Eye  Pupil Width  （mm） | Samples | Left Eye  Pupil Area  （mm^2^） | Left Eye Pupil Height  （mm） | Left Eye  Pupil Width  （mm） |
| --- | --- | --- | --- | --- | --- | --- | --- |
| WPU | 587.808 | 28.754 | 26.028 | WPU | 568.488 | 28.96 | 24.994 |
| WTM-1 | 615.958 | 28.418 | 27.598 | WTN-1 | 608.514 | 29.565 | 26.206 |
| WTM-2 | 645.508 | 30.616 | 26.845 | WTN-2 | 626.764 | 29.786 | 26.791 |
| WTM-3 | 695.873 | 30.677 | 28.882 | WTN-3 | 668.469 | 30.330 | 28.062 |
| WTM-4 | 712.260 | 31.168 | 29.097 | WTN-4 | 744.11 | 31.573 | 30.008 |
| WTM-5 | 674.415 | 29.772 | 28.842 | WTN-5 | 694.104 | 30.868 | 28.630 |
| WPU | 517.503 | 27.092 | 24.321 | WPU | 579.396 | 28.573 | 25.819 |
| WCT-1 | 561.963 | 28.546 | 25.065 | WCC-1 | 611.958 | 29.707 | 26.229 |
| WCT-2 | 622.152 | 28.894 | 27.416 | WCC-2 | 641.723 | 29.499 | 27.698 |
| WCT-3 | 640.251 | 29.407 | 27.721 | WCC-3 | 671.236 | 30.218 | 28.283 |
| WCT-4 | 688.087 | 31.579 | 27.743 | WCC-4 | 685.313 | 30.700 | 28.423 |
| WCT-5 | 555.565 | 27.284 | 25.926 | WCC-5 | 590.592 | 29.018 | 25.913 |

**Table S3.** Raw data from the left eye of the test subject during the viewing of Cherrywood samples.

| Samples | Left Eye  Pupil Area  （mm^2^） | Left Eye Pupil Height  （mm） | Left Eye  Pupil Width  （mm） | Samples | Left Eye  Pupil Area  （mm^2^） | Left Eye Pupil Height  （mm） | Left Eye  Pupil Width  （mm） |
| --- | --- | --- | --- | --- | --- | --- | --- |
| WPU | 579.427 | 29.887 | 24.685 | WPU | 583.886 | 28.062 | 26.492 |
| WTM-1 | 615.399 | 30.231 | 25.919 | WTN-1 | 616.906 | 29.723 | 26.426 |
| WTM-2 | 621.019 | 29.309 | 26.978 | WTN-2 | 676.048 | 31.364 | 27.445 |
| WTM-3 | 638.856 | 29.416 | 27.652 | WTN-3 | 694.569 | 30.754 | 28.755 |
| WTM-4 | 641.071 | 28.994 | 28.152 | WTN-4 | 720.226 | 32.446 | 28.263 |
| WTM-5 | 589.235 | 28.315 | 26.496 | WTN-5 | 653.69 | 29.269 | 28.436 |
| WPU | 586.731 | 29.133 | 25.643 | WPU | 565.973 | 28.812 | 25.011 |
| WCT-1 | 630.769 | 30.012 | 26.760 | WCC-1 | 600.916 | 29.340 | 26.078 |
| WCT-2 | 663.177 | 29.376 | 28.744 | WCC-2 | 680.631 | 31.686 | 27.349 |
| WCT-3 | 684.476 | 29.828 | 29.218 | WCC-3 | 700.136 | 31.764 | 28.065 |
| WCT-4 | 723.958 | 30.721 | 30.004 | WCC-4 | 647.129 | 30.390 | 27.112 |
| WCT-5 | 655.867 | 29.892 | 27.937 | WCC-5 | 599.664 | 29.129 | 26.202 |

**Table S4.** Raw data from the left eye of the test subject during the viewing of Sapelli samples.

| Samples | Left Eye  Pupil Area  （mm^2^） | Left Eye Pupil Height  （mm） | Left Eye  Pupil Width  （mm） | Samples | Left Eye  Pupil Area  （mm^2^） | Left Eye Pupil Height  （mm） | Left Eye  Pupil Width  （mm） |
| --- | --- | --- | --- | --- | --- | --- | --- |
| WPU | 540.265 | 27.942 | 24.618 | WPU | 502.474 | 26.978 | 23.715 |
| WTM-1 | 598.188 | 29.625 | 25.709 | WTN-1 | 545.905 | 28.653 | 24.258 |
| WTM-2 | 611.306 | 29.749 | 26.163 | WTN-2 | 598.287 | 29.139 | 26.142 |
| WTM-3 | 653.618 | 30.777 | 27.040 | WTN-3 | 641.526 | 29.592 | 27.603 |
| WTM-4 | 685.501 | 31.290 | 27.894 | WTN-4 | 693.088 | 30.262 | 29.161 |
| WTM-5 | 614.667 | 28.392 | 27.564 | WTN-5 | 574.672 | 28.207 | 25.94 |
| WPU | 546.701 | 28.544 | 24.386 | WPU | 638.216 | 30.695 | 26.473 |
| WCT-1 | 613.003 | 29.743 | 26.242 | WCC-1 | 673.245 | 30.095 | 28.483 |
| WCT-2 | 658.577 | 29.666 | 28.265 | WCC-2 | 707.735 | 30.822 | 29.236 |
| WCT-3 | 693.359 | 30.979 | 28.497 | WCC-3 | 716.744 | 31.059 | 29.382 |
| WCT-4 | 708.914 | 30.406 | 29.686 | WCC-4 | 729.062 | 30.988 | 29.956 |
| WCT-5 | 671.443 | 30.669 | 27.875 | WCC-5 | 670.377 | 29.732 | 28.708 |

**Table S5.** Parameters related to the eye tracker viewing process of Red Oak samples.

| Samples | Number of Glances | Mean Glance Duration [s] | AOI Attention Ratio [%] | Glance Location Probability [%] | PERCLOS average [%] | | Mean fixation duration left [ms] | Mean fixation duration right [ms] | Number of fixations left | Number of fixations right | Left Eye  Pupil Area  （mm^2^） |
| --- | --- | --- | --- | --- | --- | --- | --- | --- | --- | --- | --- |
| WPU | 1 | 1.197 | 8.096 | 10 | 7.209 | | 599.667 | 611 | 1 | 1 | 613.069 |
| WTM-1 | 1 | 2.016 | 9.846 | 10 | 7.209 | | 1044 | 1075 | 2 | 2 | 647.25 |
| WTM-2 | 1 | 2.448 | 11.955 | 10 | 7.209 | | 1200 | 1416 | 2 | 1 | 703.423 |
| WTM-3 | 2 | 2.768 | 13.518 | 10 | 7.209 | | 1281.5 | 1312 | 2 | 2 | 733.107 |
| WTM-4 | 3 | 2.972 | 29.224 | 50 | 7.209 | 1522.4 | | 1547.8 | 5 | 5 | 741.662 |
| WTM-5 | 1 | 3.632 | 17.738 | 10 | 7.209 | 1084.33 | | 1119.66 | 3 | 3 | 671.209 |
| WPU | 1 | 1.864 | 13.929 | 14.286 | 9.566 | 458 | | 467 | 2 | 2 | 626.474 |
| WTN-1 | 1 | 2.524 | 14.617 | 14.286 | 9.566 | 1093.5 | | 1032.5 | 2 | 2 | 643.909 |
| WTN-2 | 1 | 2.751 | 15.617 | 14.286 | 9.566 | 1165.5 | | 1177 | 2 | 2 | 662.365 |
| WTN-3 | 1 | 2.896 | 16.084 | 14.286 | 9.566 | 1254.5 | | 1243.5 | 2 | 2 | 713.244 |
| WTN-4 | 2 | 2.924 | 25.523 | 28.571 | 9.566 | 1315 | | 1345.5 | 9 | 9 | 736.364 |
| WTN-5 | 1 | 2.628 | 14.24 | 14.286 | 9.566 | 667.5 | | 892 | 4 | 3 | 658.323 |
| WPU | 1 | 1.112 | 10.33 | 11.111 | 9.869 | 332.75 | | 346.75 | 2 | 2 | 638.194 |
| WCT-1 | 2 | 1.76 | 11.435 | 11.111 | 9.869 | 349.51 | | 358.42 | 4 | 4 | 675.964 |
| WCT-2 | 1 | 2.376 | 16.513 | 11.111 | 9.869 | 792.25 | | 635.2 | 4 | 5 | 706.352 |
| WCT-3 | 1 | 2.492 | 19.721 | 22.222 | 9.869 | 991 | | 979.5 | 2 | 2 | 726.005 |
| WCT-4 | 3 | 2.659 | 24.338 | 33.333 | 9.869 | 1021.31 | | 1006.21 | 9 | 9 | 742.133 |
| WCT-5 | 1 | 1.953 | 21.338 | 11.111 | 9.869 | 735.167 | | 894.6 | 6 | 5 | 651.019 |
| WPU | 1 | 1.856 | 9.062 | 14.286 | 9.976 | 846 | | 851 | 2 | 2 | 542.07 |
| WCC-1 | 1 | 1.924 | 6.953 | 14.286 | 9.976 | 915.5 | | 937 | 2 | 2 | 608.998 |
| WCC-2 | 1 | 2.332 | 14.734 | 14.286 | 9.976 | 1066.333 | | 1128 | 3 | 2 | 627.184 |
| WCC-3 | 1 | 2.692 | 17.609 | 14.286 | 9.976 | 1281.667 | | 1296 | 3 | 3 | 650.038 |
| WCC-4 | 2 | 2.936 | 26.858 | 28.571 | 9.976 | 1682.4 | | 1717.8 | 5 | 5 | 706.817 |
| WCC-5 | 1 | 2.254 | 25.389 | 14.286 | 9.976 | 966.667 | | 961.333 | 3 | 3 | 631.862 |

**Table S6.** Parameters related to the eye tracker viewing process of European Ash samples.

| Samples | Number of Glances | Mean Glance Duration [s] | AOI Attention Ratio [%] | Glance Location Probability [%] | PERCLOS average [%] | Mean fixation duration left [ms] | Mean fixation duration right [ms] | Number of fixations left | Number of fixations right | Left Eye  Pupil Area  （mm^2^） |
| --- | --- | --- | --- | --- | --- | --- | --- | --- | --- | --- |
| WPU | 1 | 1.536 | 7.512 | 9.091 | 7.541 | 684.5 | 673 | 2 | 2 | 587.808 |
| WTM-1 | 1 | 1.632 | 11.964 | 9.091 | 7.541 | 1027 | 1047.66 | 3 | 3 | 615.958 |
| WTM-2 | 2 | 1.972 | 12.443 | 18.182 | 7.541 | 1192.75 | 1194.5 | 4 | 4 | 645.508 |
| WTM-3 | 2 | 2.215 | 14.634 | 18.182 | 7.541 | 1376 | 1390 | 2 | 2 | 695.873 |
| WTM-4 | 3 | 2.992 | 20.747 | 27.273 | 7.541 | 1594.6 | 1619 | 5 | 5 | 712.26 |
| WTM-5 | 2 | 2.736 | 16.763 | 18.182 | 7.541 | 715.571 | 628 | 7 | 8 | 674.415 |
| WPU | 1 | 1.016 | 9.905 | 7.143 | 7.561 | 642.333 | 650 | 3 | 3 | 568.488 |
| WTN-1 | 2 | 2.032 | 11.81 | 14.286 | 7.561 | 710.833 | 727.167 | 6 | 6 | 608.514 |
| WTN-2 | 2 | 2.72 | 13.259 | 14.286 | 7.561 | 1083 | 1099 | 1 | 1 | 626.764 |
| WTN-3 | 3 | 2.752 | 15.997 | 21.429 | 7.561 | 1546.5 | 1546.75 | 4 | 4 | 668.469 |
| WTN-4 | 4 | 2.908 | 27.453 | 28.571 | 7.561 | 1821 | 1842.33 | 9 | 9 | 744.11 |
| WTN-5 | 2 | 2.249 | 11.777 | 14.286 | 7.561 | 1191 | 1201 | 2 | 2 | 694.104 |
| WPU | 1 | 1.264 | 12.265 | 7.143 | 7.082 | 756.667 | 759 | 3 | 3 | 517.503 |
| WCT-1 | 1 | 1.461 | 18.073 | 14.286 | 7.082 | 806 | 837 | 2 | 2 | 561.963 |
| WCT-2 | 2 | 1.634 | 19.205 | 14.286 | 7.082 | 982 | 993.5 | 4 | 4 | 622.152 |
| WCT-3 | 3 | 1.825 | 21.644 | 21.429 | 7.082 | 1026.25 | 1036.25 | 4 | 4 | 640.251 |
| WCT-4 | 3 | 1.984 | 28.876 | 21.429 | 7.082 | 1092 | 1121.4 | 5 | 5 | 688.087 |
| WCT-5 | 3 | 1.109 | 16.146 | 21.429 | 7.082 | 798.5 | 538.333 | 4 | 6 | 555.565 |
| WPU | 2 | 1.36 | 10.303 | 16.667 | 7.949 | 587.75 | 602.25 | 4 | 4 | 579.396 |
| WCC-1 | 2 | 1.976 | 11.547 | 16.667 | 7.949 | 678.333 | 680.333 | 3 | 3 | 611.958 |
| WCC-2 | 2 | 2.216 | 12.894 | 16.667 | 7.949 | 1188.5 | 1199.5 | 2 | 2 | 641.723 |
| WCC-3 | 2 | 2.376 | 13.459 | 16.667 | 7.949 | 1155.667 | 1183.75 | 3 | 4 | 671.236 |
| WCC-4 | 3 | 2.816 | 21.763 | 16.667 | 7.949 | 1482 | 1460.75 | 5 | 4 | 685.313 |
| WCC-5 | 2 | 2.728 | 20.684 | 16.667 | 7.949 | 1027.2 | 1306.5 | 5 | 4 | 590.592 |

**Table S7.** Parameters related to the eye tracker viewing process of Cherrywood samples.

| Samples | Number of Glances | Mean Glance Duration [s] | AOI Attention Ratio [%] | Glance Location Probability [%] | PERCLOS average [%] | Mean fixation duration left [ms] | Mean fixation duration right [ms] | Number of fixations left | Number of fixations right | Left Eye  Pupil Area  （mm^2^） |
| --- | --- | --- | --- | --- | --- | --- | --- | --- | --- | --- |
| WPU | 2 | 1.192 | 9.606 | 6.667 | 7.541 | 564.2 | 587.8 | 1 | 1 | 579.427 |
| WTM-1 | 2 | 1.368 | 13.41 | 13.333 | 7.541 | 597 | 503 | 3 | 4 | 615.399 |
| WTM-2 | 2 | 1.405 | 15.116 | 13.333 | 7.541 | 616.667 | 676.75 | 1 | 4 | 621.019 |
| WTM-3 | 2 | 1.488 | 17.293 | 13.333 | 7.541 | 1058 | 1067 | 4 | 1 | 638.856 |
| WTM-4 | 3 | 1.948 | 21.35 | 20 | 7.541 | 1507.83 | 1532 | 6 | 6 | 641.071 |
| WTM-5 | 5 | 1.117 | 17.369 | 33.333 | 7.541 | 702.857 | 705.286 | 10 | 7 | 589.235 |
| WPU | 1 | 1.104 | 11.127 | 7.692 | 7.561 | 673.75 | 551.2 | 4 | 5 | 583.886 |
| WTN-1 | 2 | 1.832 | 12.109 | 15.385 | 7.561 | 766.667 | 795.333 | 2 | 3 | 616.906 |
| WTN-2 | 2 | 1.901 | 18.012 | 15.385 | 7.561 | 898.167 | 800.833 | 2 | 6 | 676.048 |
| WTN-3 | 2 | 2.064 | 19.957 | 15.385 | 7.561 | 908.25 | 947.25 | 2 | 4 | 694.569 |
| WTN-4 | 4 | 2.412 | 21.727 | 30.769 | 7.561 | 1018.33 | 1034.167 | 6 | 6 | 720.226 |
| WTN-5 | 2 | 1.816 | 17.7 | 15.385 | 7.561 | 676.2 | 677.8 | 12 | 5 | 653.69 |
| WPU | 1 | 1.68 | 8.162 | 6.667 | 7.082 | 258.4 | 276 | 4 | 5 | 586.731 |
| WCT-1 | 2 | 1.736 | 15.208 | 6.667 | 7.082 | 334.333 | 338 | 5 | 3 | 630.769 |
| WCT-2 | 3 | 1.869 | 16.67 | 13.333 | 7.082 | 481.4 | 610.25 | 2 | 4 | 663.177 |
| WCT-3 | 4 | 1.872 | 19.094 | 20 | 7.082 | 916.5 | 1164 | 4 | 1 | 684.476 |
| WCT-4 | 5 | 1.998 | 26.661 | 33.333 | 7.082 | 1035.45 | 1045.818 | 6 | 11 | 723.958 |
| WCT-5 | 3 | 1.252 | 24.449 | 20 | 7.082 | 1091 | 925.667 | 6 | 6 | 655.867 |
| WPU | 1 | 1.432 | 11.802 | 6.25 | 7.949 | 399.25 | 417.75 | 4 | 2 | 565.973 |
| WCC-1 | 2 | 1.564 | 12.705 | 12.5 | 7.949 | 599.25 | 517.75 | 2 | 4 | 600.916 |
| WCC-2 | 2 | 2.048 | 13.171 | 12.5 | 7.949 | 929.5 | 677.667 | 5 | 3 | 680.631 |
| WCC-3 | 2 | 2.208 | 15.724 | 12.5 | 7.949 | 1023 | 1046.75 | 3 | 4 | 700.136 |
| WCC-4 | 4 | 2.98 | 29.023 | 31.25 | 7.949 | 1432.5 | 1444.875 | 11 | 8 | 647.129 |
| WCC-5 | 5 | 1.954 | 23.138 | 25 | 7.949 | 568.75 | 915.2 | 3 | 5 | 599.664 |

**Table S8.** Parameters related to the eye tracker viewing process of Sapelli samples.

| Samples | Number of Glances | Mean Glance Duration [s] | AOI Attention Ratio [%] | Glance Location Probability [%] | PERCLOS average [%] | | Mean fixation duration left [ms] | Mean fixation duration right [ms] | Number of fixations left | Number of fixations right | Left Eye  Pupil Area  （mm^2^） |
| --- | --- | --- | --- | --- | --- | --- | --- | --- | --- | --- | --- |
| WPU | 1 | 1.296 | 7.137 | 9.091 | 7.433 | | 681.25 | 557.2 | 1 | 1 | 540.265 |
| WTM-1 | 1 | 1.616 | 12.031 | 9.091 | 7.433 | | 786 | 799 | 3 | 2 | 598.188 |
| WTM-2 | 1 | 1.892 | 12.89 | 9.091 | 7.433 | 861 | | 879.5 | 1 | 2 | 611.306 |
| WTM-3 | 2 | 1.987 | 13.482 | 18.182 | 7.433 | 1020.5 | | 1007.25 | 4 | 4 | 653.618 |
| WTM-4 | 3 | 2.013 | 18.84 | 27.273 | 7.433 | 1439 | | 1545.8 | 6 | 5 | 685.501 |
| WTM-5 | 3 | 2.205 | 16.942 | 27.273 | 7.433 | 829.1 | | 850 | 10 | 10 | 614.667 |
| WPU | 1 | 1.536 | 7.482 | 9.091 | 9.52 | 338 | | 350.75 | 4 | 4 | 502.474 |
| WTN-1 | 2 | 1.928 | 9.04 | 9.091 | 9.52 | 909.5 | | 914 | 2 | 2 | 545.905 |
| WTN-2 | 2 | 2.176 | 10.599 | 9.091 | 9.52 | 990 | | 1029.5 | 2 | 2 | 598.287 |
| WTN-3 | 2 | 2.44 | 17.014 | 18.182 | 9.52 | 1085.5 | | 1025 | 2 | 2 | 641.526 |
| WTN-4 | 3 | 2.521 | 19.834 | 27.273 | 9.52 | 1501.5 | | 1520.33 | 6 | 6 | 693.088 |
| WTN-5 | 3 | 2.171 | 13.41 | 27.273 | 9.52 | 652.75 | | 703.545 | 12 | 11 | 574.672 |
| WPU | 1 | 1.144 | 10.432 | 6.667 | 8.431 | 404 | | 408 | 4 | 4 | 546.701 |
| WCT-1 | 2 | 1.651 | 12.498 | 13.333 | 8.431 | 535.4 | | 445 | 5 | 4 | 613.003 |
| WCT-2 | 3 | 1.808 | 13.809 | 13.333 | 8.431 | 991.5 | | 981 | 2 | 2 | 658.577 |
| WCT-3 | 3 | 1.916 | 19.887 | 20 | 8.431 | 1010.75 | | 1019.75 | 4 | 4 | 693.359 |
| WCT-4 | 3 | 1.568 | 22.888 | 26.667 | 8.431 | 1689 | | 1606 | 6 | 7 | 708.914 |
| WCT-5 | 4 | 1.372 | 21.703 | 20 | 8.431 | 829.667 | | 718.857 | 6 | 7 | 671.443 |
| WPU | 1 | 1.752 | 11.022 | 10.526 | 10.344 | 514.25 | | 519.75 | 4 | 4 | 638.216 |
| WCC-1 | 2 | 1.712 | 16.957 | 10.526 | 10.344 | 696 | | 692 | 2 | 2 | 673.245 |
| WCC-2 | 2 | 2.584 | 17.477 | 15.789 | 10.344 | 906.8 | | 1015 | 5 | 3 | 707.735 |
| WCC-3 | 3 | 2.821 | 18.038 | 15.789 | 10.344 | 1009 | | 812.333 | 3 | 3 | 716.744 |
| WCC-4 | 6 | 2.941 | 33.455 | 31.579 | 10.344 | 1555.182 | | 1625.3 | 11 | 10 | 729.062 |
| WCC-5 | 3 | 1.661 | 19.693 | 15.789 | 10.344 | 620.667 | | 640 | 3 | 3 | 670.377 |

**Table S9.** Relevant parameters during the process of viewing the optimal combination sample using an eye tracker of Red Oak.

| Samples | Number of Glances | Mean Glance Duration [s] | AOI Attention Ratio [%] | Glance Location Probability [%] | PERCLOS average [%] | Mean fixation duration left [ms] | Mean fixation duration right [ms] | Number of fixations left | Number of fixations right | Left Eye  Pupil Area  （mm^2^） |
| --- | --- | --- | --- | --- | --- | --- | --- | --- | --- | --- |
| WPU | 3 | 1.801 | 20.365 | 18.182 | 7.082 | 581.427 | 601.753 | 6 | 6 | 661.475 |
| WTM-4 | 2 | 1.542 | 16.175 | 16.067 | 7.082 | 510.893 | 525.417 | 4 | 4 | 641.583 |
| WTN-4 | 1 | 1.218 | 10.622 | 9.091 | 7.082 | 498.347 | 513.723 | 2 | 2 | 611.547 |
| WCT-4 | 4 | 2.154 | 24.587 | 23.327 | 7.082 | 754.872 | 832.854 | 7 | 7 | 689.214 |
| WCC-4 | 5 | 2.637 | 28.251 | 33.333 | 7.082 | 956.241 | 997.487 | 10 | 10 | 705.342 |

**Table S10.** Relevant parameters during the process of viewing the optimal combination sample using an eye tracker of European Ash.

| Samples | Number of Glances | Mean Glance Duration [s] | AOI Attention Ratio [%] | Glance Location Probability [%] | PERCLOS average [%] | Mean fixation duration left [ms] | Mean fixation duration right [ms] | Number of fixations left | Number of fixations right | Left Eye  Pupil Area  （mm^2^） |
| --- | --- | --- | --- | --- | --- | --- | --- | --- | --- | --- |
| WPU | 3 | 1.802 | 20.487 | 19.631 | 7.433 | 571.624 | 591.421 | 5 | 5 | 658.973 |
| WTM-4 | 1 | 1.492 | 14.175 | 12.857 | 7.433 | 509.125 | 519.641 | 3 | 3 | 631.474 |
| WTN-4 | 1 | 1.114 | 9.74 | 10.527 | 7.433 | 492.542 | 509.421 | 1 | 1 | 603.743 |
| WCT-4 | 4 | 2.212 | 25.634 | 21.749 | 7.433 | 781.674 | 801.649 | 8 | 8 | 690.613 |
| WCC-4 | 5 | 2.732 | 29.964 | 35.236 | 7.433 | 893.645 | 897.899 | 11 | 11 | 713.843 |

**Table S11.** Relevant parameters during the process of viewing the optimal combination sample using an eye tracker of Cherrywood.

| Samples | Number of Glances | Mean Glance Duration [s] | AOI Attention Ratio [%] | Glance Location Probability [%] | PERCLOS average [%] | Mean fixation duration left [ms] | Mean fixation duration right [ms] | Number of fixations left | Number of fixations right | Left Eye  Pupil Area  （mm^2^） |
| --- | --- | --- | --- | --- | --- | --- | --- | --- | --- | --- |
| WPU | 3 | 2.053 | 18.665 | 18.451 | 7.561 | 582.311 | 601.287 | 6 | 6 | 663.187 |
| WTM-4 | 2 | 1.872 | 15.096 | 11.993 | 7.561 | 529.411 | 543.901 | 4 | 4 | 629.693 |
| WTN-4 | 2 | 1.531 | 11.437 | 11.941 | 7.561 | 501.235 | 510.258 | 2 | 2 | 605.738 |
| WCT-4 | 4 | 2.474 | 24.468 | 22.631 | 7.561 | 802.472 | 810.473 | 9 | 9 | 701.329 |
| WCC-4 | 6 | 2.891 | 30.334 | 34.984 | 7.561 | 901.582 | 911.345 | 12 | 12 | 725.384 |

**Table S12.** Relevant parameters during the process of viewing the optimal combination sample using an eye tracker of Sapelli.

| Samples | Number of Glances | Mean Glance Duration [s] | AOI Attention Ratio [%] | Glance Location Probability [%] | PERCLOS average [%] | Mean fixation duration left [ms] | Mean fixation duration right [ms] | Number of fixations left | Number of fixations right | Left Eye  Pupil Area  （mm^2^） |
| --- | --- | --- | --- | --- | --- | --- | --- | --- | --- | --- |
| WPU | 4 | 2.518 | 17.899 | 19.836 | 7.949 | 738.386 | 751.492 | 7 | 7 | 651.479 |
| WTM-4 | 2 | 2.013 | 15.209 | 15.098 | 7.949 | 653.896 | 687.981 | 5 | 5 | 610.683 |
| WTN-4 | 1 | 1.894 | 14.227 | 9.855 | 7.949 | 559.217 | 588.042 | 1 | 1 | 598.472 |
| WCT-4 | 5 | 2.892 | 22.778 | 25.095 | 7.949 | 812.638 | 837.931 | 10 | 10 | 699.954 |
| WCC-4 | 6 | 3.012 | 29.887 | 30.116 | 7.949 | 890.654 | 901.418 | 11 | 11 | 708.343 |

**References**

[1] Budde, W.J. Metrologia, A Reference Instrument for 20°, 60° and 85° Gloss Measurements, 16 (1980) 1-5.

[2] Standard Test Method for Film Hardness by Pencil Test, D01.23, 2011.

[3] Standard Test Method for Pull-Off Strength of Coatings Using Portable Adhesion Testers, D01.46, 2022.

[4] N. Li, J. Zhang, C. Wang, H. Sun, Enhanced photocatalytic degradation of tetrabromobisphenol A by tourmaline–TiO_2_ composite catalyst, Journal of Materials Science, 52 (2017) 6937-6949.

[5] S. Jantathai, L. Danner, M. Joechl, K. Dürrschmid, Gazing behavior, choice and color of food: Does gazing behavior predict choice?, Food Research International, 54 (2013) 1621-1626.

[6] V.L. Deringer, A.P. Bartók, N. Bernstein, D.M. Wilkins, M. Ceriotti, G. Csányi, Gaussian Process Regression for Materials and Molecules, Chemical Reviews, 121 (2021) 10073-10141.

[7] J. Lee, D. Park, M. Lee, H. Lee, K. Park, I. Lee, S. Ryu, Machine learning-based inverse design methods considering data characteristics and design space size in materials design and manufacturing: a review, Materials Horizons, 10 (2023) 5436-5456.

[8] A.G. Kusne, H. Yu, C. Wu, H. Zhang, J. Hattrick-Simpers, B. DeCost, S. Sarker, C. Oses, C. Toher, S. Curtarolo, A.V. Davydov, R. Agarwal, L.A. Bendersky, M. Li, A. Mehta, I. Takeuchi, On-the-fly closed-loop materials discovery via Bayesian active learning, Nature Communications, 11 (2020) 5966.
